# Expanding the targeting scope of CRISPR/Cas9-mediated genome editing by Cas9 variants in *Brassica*

**DOI:** 10.1007/s42994-024-00155-7

**Published:** 2024-04-05

**Authors:** Wenjing Li, Xuan Li, Chunyang Wang, Guanzhong Huo, Xinru Zhang, Jintai Yu, Xiaoxiao Yu, Jing Li, Chao Zhang, Jianjun Zhao, Yan Li, Jun Li

**Affiliations:** 1 State Key Laboratory of North China Crop Improvement and Regulation, College of Life Sciences, Hebei Agricultural University, Baoding, 071001 China; 2 Hebei Key Laboratory of Plant Physiology and Molecular Pathology, Hebei Agricultural University, Baoding, 071001 China; 3 Key Laboratory of Vegetable Germplasm Innovation and Utilization of Hebei, Collaborative Innovation Center of Vegetable Industry in Hebei, College of Horticulture, Hebei Agricultural University, Baoding, 071001 China; 4 College of Modern Science and Technology, Hebei Agricultural University, Baoding, 071001 China

**Keywords:** *Brassica*, Cas9 variants, CRISPR, Genome editing, Non-canonical PAM

## Abstract

CRISPR/Cas9, presently the most widely used genome editing technology, has provided great potential for functional studies and plant breeding. However, the strict requirement for a protospacer adjacent motif (PAM) has hindered the application of the CRISPR/Cas9 system because the number of targetable genomic sites is limited. Recently, the engineered variants Cas9-NG, SpG, and SpRY, which recognize non-canonical PAMs, have been successfully tested in plants (mainly in rice, a monocot). In this study, we evaluated the targeted mutagenesis capabilities of these Cas9 variants in two important *Brassica* vegetables, Chinese cabbage (*Brassica rapa* spp. *pekinensis*) and cabbage (*Brassica oleracea* var. *capitata*). Both Cas9-NG and SpG induced efficient mutagenesis at NGN PAMs, while SpG outperformed Cas9-NG at NGC and NGT PAMs. SpRY achieved efficient editing at almost all PAMs (NRN > NYN), albeit with some self-targeting activity at transfer (T)-DNA sequences. And SpRY-induced mutants were detected in cabbage plants in a PAM-less fashion. Moreover, an adenine base editor was developed using SpRY and TadA8e deaminase that induced A-to-G conversions within target sites using non-canonical PAMs. Together, the toolboxes developed here induced successful genome editing in Chinese cabbage and cabbage. Our work further expands the targeting scope of genome editing and paves the way for future basic research and genetic improvement in *Brassica*.

**Supplementary Information:**

The online version contains supplementary material available at 10.1007/s42994-024-00155-7.

Dear Editor,

Chinese cabbage (*Brassica rapa* spp. *pekinensis*) and cabbage (*Brassica oleracea* var. *capitata*), of the genus *Brassica*, are major cultivated vegetable species worldwide (Li et al. 2022). They are exceptional sources of vitamins and minerals; thus, they are critical to food and nutritional security. Due to a whole-genome triplication event that affected all species in the genus, there are several multicopy genes and significantly related homologous genes in *Brassica* (Cai et al. 2021). The complex polyploid nature of *Brassica* species has hindered functional genomics and breeding programs for Chinese cabbage and cabbage.

The CRISPR/Cas9 system, which originated as a prokaryotic adaptive immune system, has sparked a revolution in gene editing and is currently widely used for genome modification in various species, including those in the genus *Brassica* (Li et al. 2021b, 2022; Molla et al. 2021). It has great applications and advances in functional studies and genetic improvement. CRISPR/Cas9 includes two components, the Cas9 endonuclease and a chimeric single guide RNA (sgRNA). Target recognition and cleavage by CRISPR/Cas9 depends not only on complementarity between the sgRNA and target DNA but also on the existence of a protospacer adjacent motif (PAM) located immediately downstream of the target site. The most robust and widely used Cas9, which originated in *Streptococcus pyogenes* (SpCas9), uses NGG as the canonical PAM (Walton et al. 2020). However, the requirement for a PAM has constrained the targeting scope and flexibility of CRISPR/Cas9 and CRISPR/Cas9-based base editing (Li et al. 2020; Ren et al. 2021a; Wei et al. 2021).

To expand the targeting scope of genome editing, several Cas endonucleases and Cas9 variants with distinct PAM preferences have been developed (Walton et al. 2020; Molla et al. 2021). For example, gene-editing and base editing by three engineered SpCas9 variants, Cas9-NG and SpG, which recognize relaxed NGN PAMs, and SpRY, which targets almost all PAMs, have been reported mainly in monocotyledonous plants (Li et al. 2021a; Ren et al. 2021a, b; Xu et al. 2021). However, whether these engineered Cas9 variants function well in *Brassica* species is unknown.

We conducted an in silico PAM analysis of the genomes of Chinese cabbage and cabbage to assess the potential targetable range of SpCas9, Cas9-NG, SpG, and SpRY in *Brassica*. A computer search revealed that SpCas9 could theoretically target 11.97% and 10.44% of genome sites in Chinese cabbage and cabbage, respectively. Cas9-NG/SpG could potentially target at least twice as many sites in the genome than SpCas9 (Fig. S1 and Table S1). Theoretically, SpRY could target all sites with NNN PAMs. Therefore, there is a great potential to develop Cas9-NG, SpG, and SpRY-mediated genome editing in *Brassica* due to their promise to greatly expand the targeting scope. Considering that protoplast-based transient expression systems have been widely used to validate the activity of CRISPR vectors and to systematically optimize genome editing tools (Gao 2021; Gaillochet et al. 2023), we compared the efficiencies of the Cas9-NG, SpG, and SpRY variants in different PAM contexts using Chinese cabbage and cabbage protoplasts. If successful, it will expand the targeting scope of gene editing to a PAM-less fashion in *Brassica*.

To expand the targeting scope of CRISPR/Cas9-mediated genome editing in Chinese cabbage and cabbage, we introduced Cas9-NG and SpG (Li et al. 2021a) into pBSE401 (Xing et al. 2014), generating pBSE-Cas9-NG and pBSE-SpG, respectively (Fig. 1A). Given that Cas9-NG and SpG recognize NGN PAMs, we next constructed targets for both pBSE-Cas9-NG and pBSE-SpG using NGN PAMs and the genes *BrPDS*, *BrAOP2*, *BoPDS*, and *BoDMR6* (Table S2). The targeting vectors were transformed into protoplasts, and the genome editing efficiencies of Cas9-NG and SpG were quantified based on next-generation sequencing (NGS) of PCR amplicons. At NGN PAMs, Cas9-NG showed efficient editing activity, with insertion/deletion (indel) mutation frequencies of 2.12–8.56% (Fig. 1B and Fig. S2). SpG also exhibited robust editing activity, with indel mutation frequencies of 1.92–15.29% (Fig. 1C and Fig. S3). These results indicate that genomic loci have effects on the editing activity of Cas9-NG and SpG. We also did a side-by-side comparison of the nuclease activities of Cas9-NG and SpG using the same sgRNAs. On average, SpG had a higher editing efficiency (1.67- to 2.79-fold greater) than Cas9-NG at NGT and NGC PAMs (Fig. 1D and E), consistent with previous findings in human cells and rice (Walton et al. 2020; Li et al. 2021a). Whereas there was no significant difference in editing efficiency between SpG and Cas9-NG at NGA PAM (Fig. 1E). Collectively, both Cas9-NG and SpG were able to recognize a broad range of NGN PAMs in *Brassica*.

**Fig. 1:**
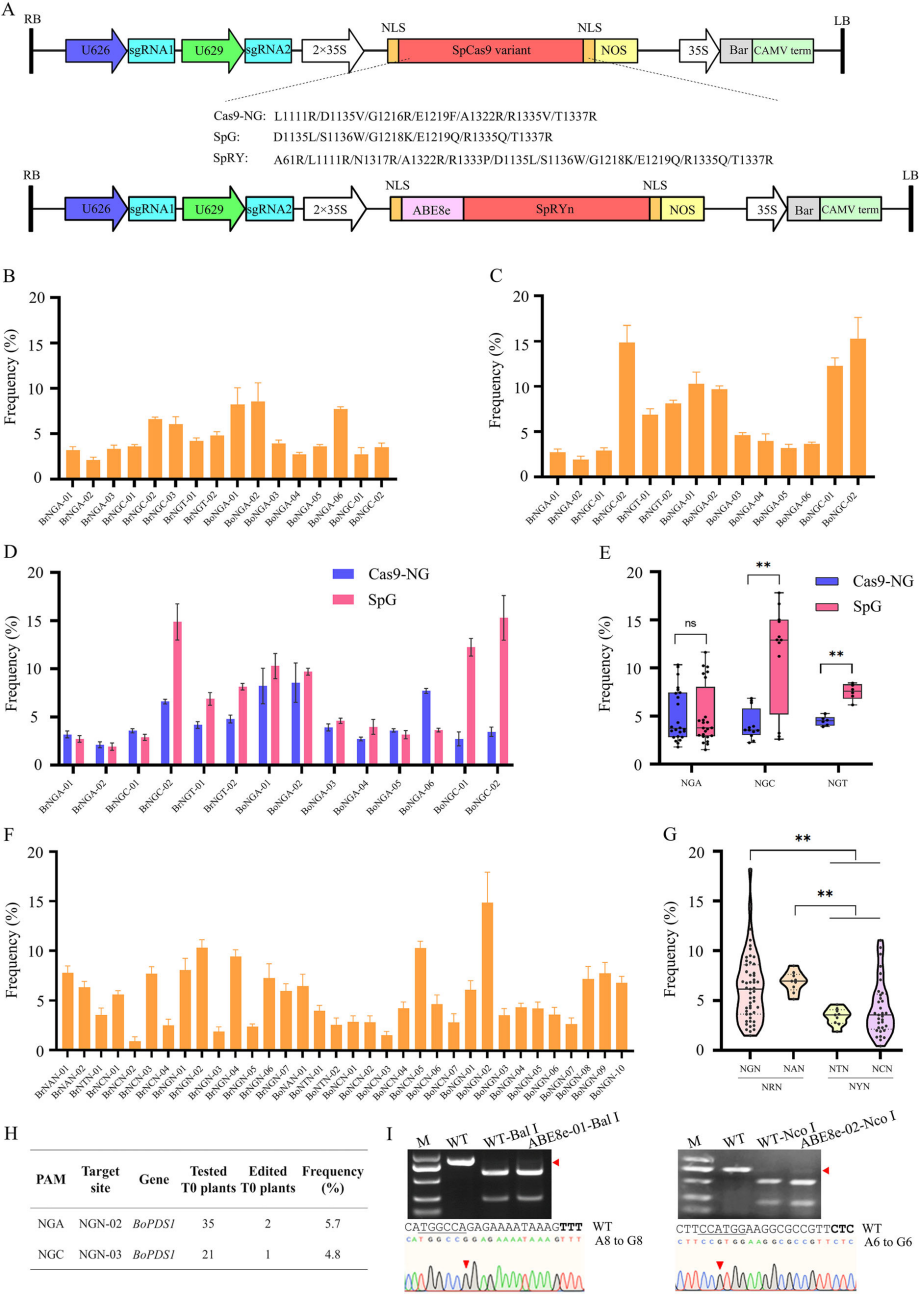
Genome editing in Chinese cabbage and cabbage using the SpCas9 variants Cas9-NG, SpG, and SpRY. **A** Schematics of the four SpCas9 variant-based genome editing tools. **B** Frequencies of targeted mutagenesis by Cas9-NG at NGN PAMs (three biological replicates). **C** Frequencies of targeted mutagenesis by SpG at NGN PAMs (three biological replicates). **D** Comparison of the targeted mutagenesis efficiencies of Cas9-NG and SpG using the same sgRNAs. **E** Comparison of the overall mutation efficiencies of Cas9-NG and SpG at the same targets with NGA, NGC, and NGT PAMs. **F** SpRY-mediated genome editing at 34 targets with NNN PAMs (three biological replicates). **G** Assessment of the preference of SpRY-mediated mutagenesis for the second N in NNN PAMs. Each dot represents a biological replicate. **H** SpRY-induced mutants in T0 plants. **I** PCR/RE assay to detect SpRYn-ABE8e-mediated adenine base editing. Sanger sequencing chromatograms of ABE8es-mediated A-to-G substitution are shown. The G base resulting from the editing was marked in a red arrow

To further expand the targeting scope of the CRISPR/Cas9 system, the nuclease SpRY was introduced into pBSE401, generating pBSE-SpRY (Fig. 1A). Following the experimental procedure described above, the genome editing efficiency of pBSE-SpRY at 34 targets bearing NNN PAMs was determined based on NGS of Chinese cabbage and cabbage protoplast amplicons (Supplementary Table 2). The sequence data indicate that SpRY performed well at NGN targets, with an efficiency ranging from 1.92 to 14.95% (Fig. 1F and G, Fig. S4). It also displayed an acceptable editing efficiency at NAN targets, with values ranging from 6.37 to 7.78% (Fig. 1F and G). At NYN (N = C/T) targets, the efficiency values are lower, with a range of 0.92–10.33% (Fig. 1G). Consistent with recent data from human cells and rice (Walton et al. 2020; Li et al. 2021a), SpRY exhibited relatively greater editing efficiency at NRN (R = G/A) sites than at NYN sites in *Brassica*. Collectively, our data show that SpRY is capable of targeting near-PAM-less (NRN > NYN) sites, albeit with variable efficiency.

To assess the activity of SpRY *in planta*, binary vectors containing sgRNAs specific to the six targets with NNN PAMs were transformed into the cabbage by *Agrobacterium*-mediated transformation (Supplementary Fig. 5). Genomic DNA was extracted from young leaf cells of Basta-resistant T0 plants. The target regions were amplified by PCR and analyzed individually using PCR/restriction enzyme (RE) assays and Sanger sequencing. Mutagenesis with an efficiency of 5.7% and 4.8% was detected at the NGN-02 and NGN-03 target, respectively (Fig. 1H, Fig. S5). Considering the high gene editing efficiency of SpRY achieved in protoplasts, the protocol of *Agrobacterium*-mediated genetic transformation for cabbage needs to be improved in our lab. Together, our findings demonstrate that SpRY was able to perform targeted mutagenesis in cabbage plants in a PAM-less fashion.

To introduce precise adenine base editing at near-PAM-less sites, SpRY(D10A) nickase was fused to TadA8e, a recently evolved highly compatible adenosine deaminase (Richter et al. 2020), resulting in pBSE-SpRYn-ABE8e (Fig. 1A). The editing window of ABE8e is typically at positions 4–8 (Richter et al. 2020). Two targets containing RE sites with TTT and CTC PAMs were constructed in pBSE-SpRYn-ABE8e, and target A was at positions 8 and 6 of the target sites, respectively (Table S2). The vectors were then transformed into protoplasts. A-to-G substitutions were identified at both sites through PCR/RE assays and Sanger sequencing, with frequencies of 7–10% (Fig. 1I). Our data show that SpRYn-ABE8e could induce adenine base editing at relaxed PAMs in Chinese cabbage.

The broadened PAM compatibility of these variants raises questions about off-target effects during genome editing. To alleviate this effect in *Brassica*, fidelity-enhanced substitutions such as SpCas9-HF1 or eSpCas9 could be introduced into these variants as reported in human cells and soybean (Walton et al. 2020; He et al. 2022). In addition, the careful design and selection of targets may reduce off-target cleavage. Finally, delivering preassembled CRISPR/Cas9 ribonucleoproteins (RNPs) directly into *Brassica* protoplasts/calli to achieve targeted mutagenesis in regenerated plants could be used to increase the specificity of CRISPR/Cas9.

Another potential issue with these variants is self-targeting (Qin et al. 2020; Ren et al. 2021b). Fourteen PAMs for Cas9-NG, SpG, and SpRY were examined for their self-editing capability in protoplasts. Among these sites, one out of four Cas9-NG targets had T-DNA mutations with a self-editing frequency of 5.21%, while one out of three SpG targets had T-DNA mutations with a self-editing frequency of 3.47%. For the SpRY variant, four out of seven targets exhibited self-editing with a frequency of 5.26–45.41% (Table S3). These data indicate that self-editing is more likely to occur using Cas9 variants, but that it might be minimized by delivering RNPs directly into protoplasts/calli.

In summary, our results show that Cas9-NG and SpG achieved high genome-editing efficiencies with NGN PAMs, while SpRY performed well with almost all of the PAMs, in Chinese cabbage and cabbage. Additionally, an adenine base editor developed using SpRY and TadA8e deaminase (SpRYn-ABE8e) produced A-to-G substitutions with non-canonical PAMs in Chinese cabbage. These findings expand the targeting scope of CRISPR/Cas9-mediated genome editing and its potential applications in *Brassica*.

## Materials and methods

### Vector design and plasmid construction

The fragment Cas9-NG, obtained through the double digestion of pHUC411-NG using *Xba*I and *Sac*I (Li et al. 2021a), was subcloned downstream of the CAMV 35S promoter to replace SpCas9 in pBSE401 (Xing et al. 2014), resulting in pBSE-Cas9-NG. SpG was amplified using primers pB-*Xba*I-F1/pB-*Sac*I-R1 with pHUC-SpG as the template and then ligated with the backbone of *Xba*I/*Sac*I-digested pBSE401 via homologous recombination to produce pBSE-SpG. pBSE-SpRY was generated using a similar method. SpRYn-ABE8e was amplified using primers pB-*Xba*I-F1/pB-*Sac*I-R1 with SpRY-eABE8 as the template and then ligated with the backbone of *Xba*I/*Sac*I-digested pBSE401 via homologous recombination to produce pBSE-SpRYn-ABE8e. The vectors were all confirmed by Sanger sequencing (Sangong Biotech, Shanghai, China). The full-length nucleotide coding sequences of Cas9-NG, SpG, SpRY, and SpRYn-ABE8e are listed in Table S4.

Targets were randomly selected at different loci with appropriate PAMs. Two sgRNAs were constructed following the protocol for pBSE401 vectors (Xing et al. 2014). Two sgRNA expression cassettes were amplified using a four-primer mixture with pCBC-DT1T2 as the template and then inserted into *Bsa*I-digested pBSE-Cas9-NG, pBSE-SpG, pBSE-SpRY, or pBSE-SpRY-ABE8e by Golden Gate cloning. The sgRNA regions were confirmed by Sanger sequencing, and the activities of the two sites were tested simultaneously.

### Chinese cabbage and cabbage protoplast transformation

Seedlings of Chinese cabbage and cabbage were grown under a 16-h-light/8-h-dark cycle at 25 °C in a growth room for 2 weeks. The protocols for protoplasts isolation and transformation were as described, with slight modifications (Li et al. 2016; Zhang et al. 2023).

Healthy fresh true leaves were cut into strips and digested with the enzyme solution (1.5% Cellulase R10, 0.75% Macerozyme R10, 0.4 M mannitol, 20 mM MES, 10 mM CaCl_2_, and 0.1% bovine serum). After 6 h digestion with shaking (40–50 rpm) at 25 °C in the dark, protoplasts were isolated by filtration through a 40 μm cell strainer. The protoplast pellet was collected and washed with W5 (154 mM NaCl, 125 mM CaCl_2_, 5 mM KCl, and 2 mM MES), then resuspended to a final concentration of 1 × 10^6^ in MMG solution (4 mM MES, 0.4 M mannitol, and 15 mM MgCl_2_).

In brief, 20 μg pBSE-Cas9-NG, pBSE-SpG, or pBSE-SpRY construct was mixed with 200 µL protoplasts (2 × 10^5^ protoplasts), and then transfected with 220 µL freshly prepared PEG solution (40% PEG, 0.2 M mannitol, and 0.1 M CaCl_2_). Transfected protoplasts were incubated in 2 ml W5 solution and cultured at 25 °C in the dark. Plasmid *pEASY-35S::*GFP (Zhang et al. 2023) was used to calculate the transfection efficiency of protoplasts. The transfection efficiency was ~ 75% for Chinese cabbage and ~ 65% for cabbage.

### Amplicon deep sequencing and data analysis

For assessing mutagenesis in protoplasts, protoplasts of Chinese cabbage or cabbage were collected 48 h after transfection. About 2 × 10^5^ protoplasts were collected and combined as a single sample. Pooled protoplast genomic DNA was extracted and PCR-amplified with high-fidelity DNA polymerase. In the first round of PCR, the target region was amplified using site-specific primers and protoplast DNA as the template. In the second round, primers with added forward and reverse barcodes were used to generate 235–255 bp PCR products for library construction. Equal amounts of the PCR products were pooled and sequenced commercially (GENEWIZ, Azenta Life Sciences, South Plainfield, NJ, USA) using an Illumina HiSeqX instrument (Illumina Inc., San Diego, CA, USA). It generally generated 6,000–18,000 clean reads for each target. The target sites in the sequenced reads were examined for indels. The mutation frequency was calculated as (No. of indel-containing reads)/(total No. of reads)(protoplast transfection efficiency). Amplicon sequencing was repeated three times for each target site using genomic DNA extracted from three independent protoplast samples. The primers used in our mutation frequency analysis are listed in Table S4.

Base editing was identified by PCR/RE assay. The amplicons were digested with a restriction enzyme that recognizes wild-type target sequences. The A-to-G conversion induced by ABE8e were resistant to restriction enzyme digestion and resulted in uncleaved bands. ImageJ 1.53t software was used to obtain the intensities of bands representing uncleaved and cleaved DNA fragments. Base-editing frequency was calculated as the ratio of uncleaved bands against all bands (Sretenovic et al. 2021). Uncleaved bands were purified and cloned into the pEASY-Blunt vector (TransGen Biotech), positive clones were sequenced to obtain the mutated sequences.

### *Agrobacterium*-mediated genetic transformation of cabbage

A highly inbred line, “2n”, from our laboratory was used for genetic transformation. Cotyledon and hypocotyl transformations were performed as described previously with modifications (Bhalla and Singh 2008; Han et al. 2023). Briefly, the cotyledons (including approximately 2 mm of the petioles) and hypocotyls from 6-day-old seedlings were cut into 0.8–1-cm segments. These explants were precultured for 2 days and then inoculated and co-cultured with *Agrobacterium* cells (GV3101 containing plasmids). After co-cultivation for 2 days, the explants were transferred to a selection medium (containing 10 mg/L of Basta). Basta-resistant shoots were obtained under selection culture.

### Genome-wide analysis of PAMs in Chinese cabbage and cabbage

The genomes of *Brassica rapa* (GCF_000309985.2_CAAS_Brap_v3.01_genomic.fna) and *Brassica oleracea* (GCF_000695525.1_BOL_genomic.fna) were downloaded from the NCBI Genome Annotation Project. Using a Regular Expressions search in Python, the FASTA file was scanned line by line and split into single characters to find the corresponding PAM type and its complementary sequence. For instance, to search for NGG PAMs in a row from the cabbage genome, we used re.findall(r"[atcgATCG][gG][gG]",brap_genome) or re.findall(r"[cC][cC][atcgATCG] ",brap_genome). The total number of characters was generated using: list_split = line.split(), list_str = ".join(list_split), cols.append(list_str).

### Detection of self-editing

Seven target sites with NGN PAMs and seven target sites with NNN PAMs were selected to test the self-editing efficiency (Table S5). Primers with barcodes were designed to amplify the T-DNA region spanning the sgRNA sites using protoplast DNA as the template. The self-editing efficiency was evaluated by amplicon sequencing. The primers used in our analysis are listed in Table S4.

### Statistical analysis

GraphPad Prism 9 software was used to analyze the data. Data are presented as the mean value ± standard deviation (SD) from three independent experiments. Two-tailed Student’s *t*-test was used. *P* < 0.05 was considered significant.

### Supplementary Information

Below is the link to the electronic supplementary material.

Supplementary file1 (DOCX 966 KB)

## Data Availability

The datasets generated during and/or analysed during the current study are available from the corresponding author on reasonable request.

## References

[CR1] Bhalla PL, Singh MB (2008). *Agrobacterium*-mediated transformation of *Brassica napus* and *Brassica oleracea*. Nat Protoc.

[CR2] Cai X, Chang L, Zhang T (2021). Impacts of allopolyploidization and structural variation on intraspecific diversification in *Brassica rapa*. Genome Biol.

[CR3] Gaillochet C, Peña Fernández A, Goossens V (2023). Systematic optimization of Cas12a base editors in wheat and maize using the ITER platform. Genome Biol.

[CR4] Gao C (2021). Genome engineering for crop improvement and future agriculture. Cell.

[CR5] Han F, Yuan K, Sun W (2023). A natural mutation in the promoter of Ms-cd1 causes dominant male sterility in *Brassica oleracea*. Nat Commun.

[CR6] He R, Zhang P, Yan Y, Yu C, Jiang L, Zhu Y, Wang D (2022). Expanding the range of CRISPR/Cas9-directed genome editing in soybean. aBIOTECH.

[CR7] Li J, Meng X, Zong Y, Chen K, Zhang H, Liu J, Gao C (2016). Gene replacements and insertions in rice by intron targeting using CRISPR/Cas9. Nat Plants.

[CR8] Li J, Li H, Chen J, Yan L, Xia L (2020). Toward precision genome editing in crop plants. Mol Plant.

[CR9] Li J, Xu R, Qin R, Liu X, Kong F, Wei P (2021). Genome editing mediated by SpCas9 variants with broad non-canonical PAM compatibility in plants. Mol Plant.

[CR10] Li Y, Li W, Li J (2021). The CRISPR/Cas9 revolution continues: from base editing to prime editing in plant science. J Genet Genom.

[CR11] Li J, Yu X, Zhang C, Li N, Zhao J (2022). The application of CRISPR/Cas technologies to *Brassica* crops: current progress and future perspectives. aBIOTECH.

[CR12] Molla KA, Sretenovic S, Bansal KC, Qi Y (2021). Precise plant genome editing using base editors and prime editors. Nat Plants.

[CR13] Qin R, Li J, Liu X, Xu R, Yang J, Wei P (2020). SpCas9-NG self-targets the sgRNA sequence in plant genome editing. Nat Plants.

[CR14] Ren J, Meng X, Hu F (2021). Expanding the scope of genome editing with SpG and SpRY variants in rice. Sci China Life Sci.

[CR15] Ren Q, Sretenovic S, Liu S (2021). PAM-less plant genome editing using a CRISPR-SpRY toolbox. Nat Plants.

[CR16] Richter MF, Zhao KT, Eton E (2020). Phage-assisted evolution of an adenine base editor with improved Cas domain compatibility and activity. Nat Biotechnol.

[CR17] Sretenovic S, Yin D, Levav A, Selengut JD, Mount SM, Qi Y (2021). Expanding plant genome-editing scope by an engineered iSpyMacCas9 system that targets A-rich PAM sequences. Plant Commun.

[CR18] Walton RT, Christie KA, Whittaker MN, Kleinstiver BP (2020). Unconstrained genome targeting with near-PAMless engineered CRISPR-Cas9 variants. Science.

[CR19] Wei C, Wang C, Jia M (2021). Efficient generation of homozygous substitutions in rice in one generation utilizing an rABE8e base editor. J Integr Plant Biol.

[CR20] Xing H, Dong L, Wang Z (2014). A CRISPR/Cas9 toolkit for multiplex genome editing in plants. BMC Plant Biol.

[CR21] Xu Z, Kuang Y, Ren B (2021). SpRY greatly expands the genome editing scope in rice with highly flexible PAM recognition. Genome Biol.

[CR22] Zhang K, Liu S, Fu Y (2023). Establishment of an efficient cotton root protoplast isolation protocol suitable for single-cell RNA sequencing and transient gene expression analysis. Plant Methods.

