# Supplementary material for: Expanding the targeting scope of CRISPR/Cas9-mediated genome editing by Cas9 variants in Brassica
Source: aBIOTECH. 2024 Apr 5;5(2):202–8. doi: 10.1007/s42994-024-00155-7 (PMC11224048; doi:10.1007/s42994-024-00155-7)
Supplement: Supplementary file 1 — Supplementary file1 (DOCX 966 KB) [file 42994_2024_155_MOESM1_ESM.docx]

**
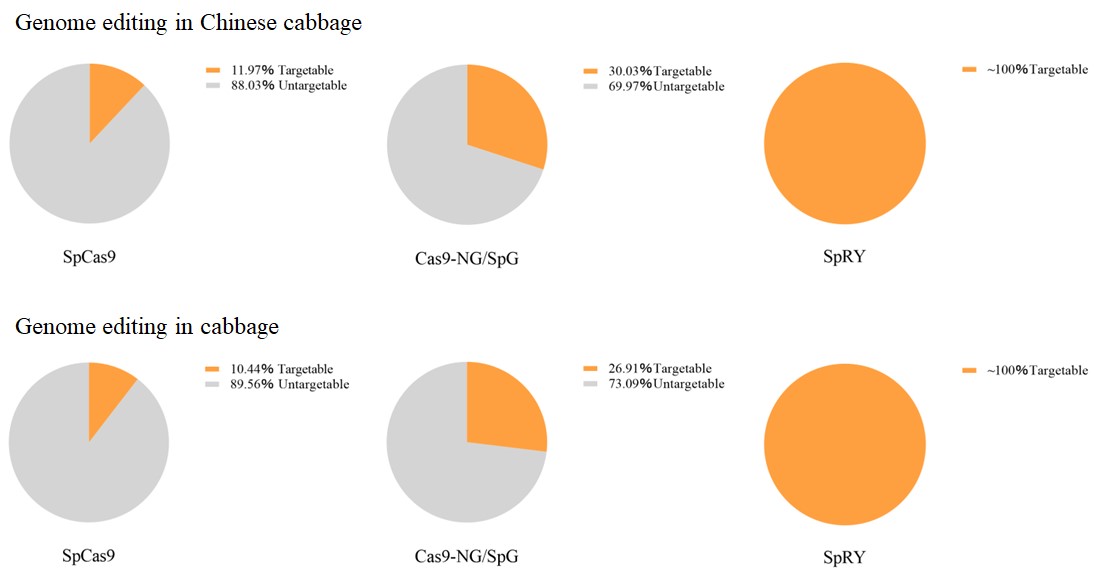
**

**Supplementary Fig. 1** Percent of targetable sites in principle by CRISPR/Cas9 genome editing technology using SpCas9, Cas9-NG, SpG, and SpRY in Chinese cabbage and cabbage. *In silico* analysis of NGG, NGN, and NNN PAMs in the genomes of Chinese cabbage and cabbage.

**
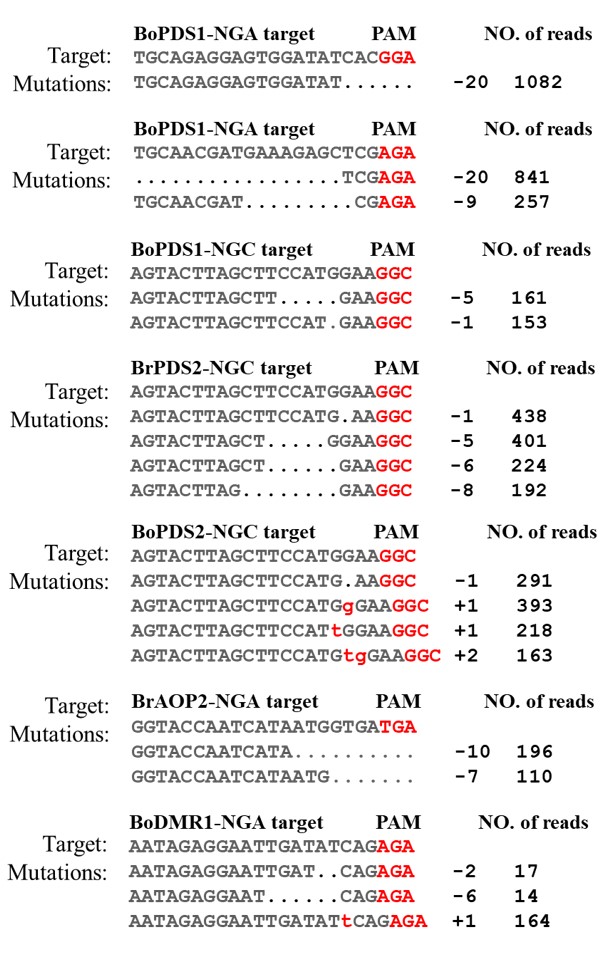
**

**Supplementary Fig. 2** Representative mutations induced by Cas9-NG in protoplasts at non-canonical PAMs. Deletions and insertions are indicated as dots and lowercase letters, respectively. Right of sequence: –/+n represents the number of base pairs that were deleted/inserted.

**
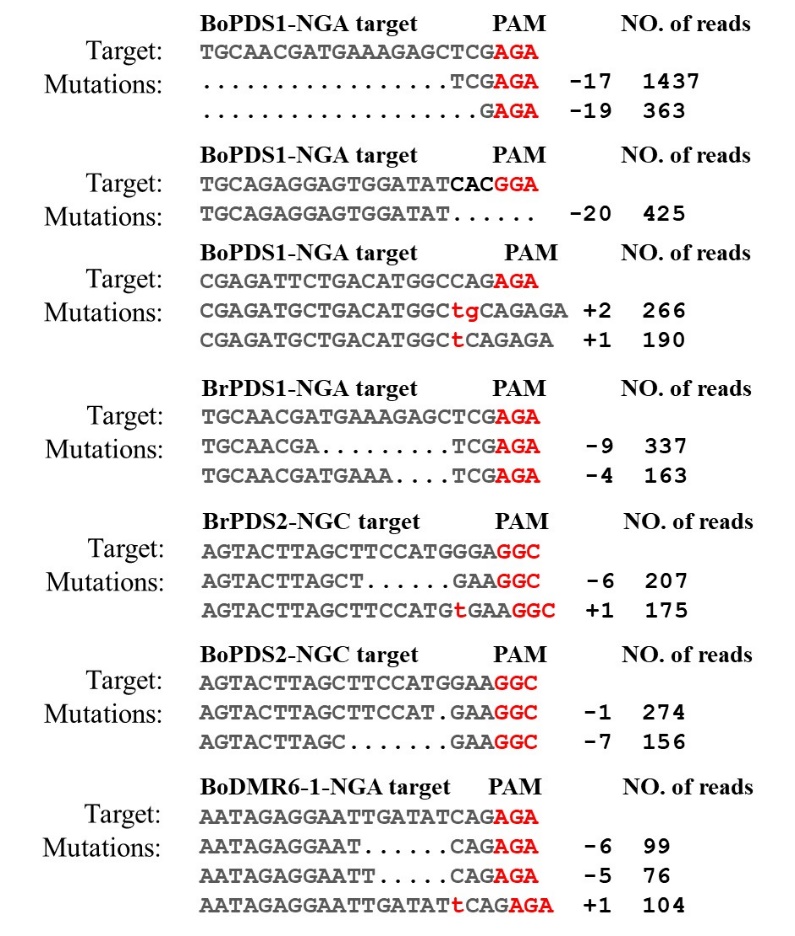
**

**Supplementary Fig. 3** Representative mutations induced by SpG in protoplasts at non-canonical PAMs. Deletions and insertions are indicated as dots and lowercase letters, respectively. Right of sequence: –/+n represents the number of base pairs that were deleted/inserted.


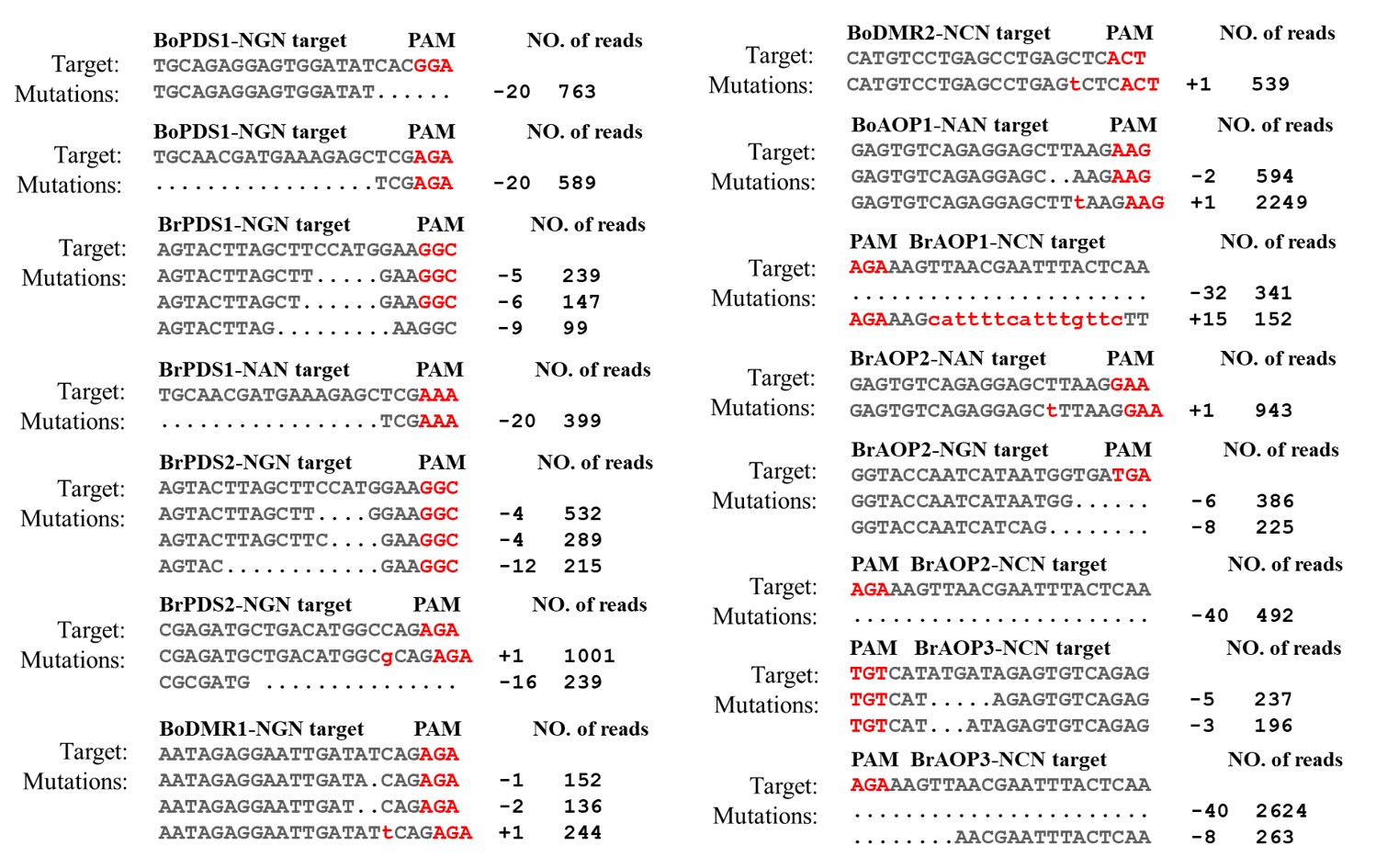


**Supplementary Fig. 4** Representative mutations induced by SpRY in protoplasts at non-canonical PAMs. Deletions and insertions are indicated as dots and lowercase letters, respectively. Right of sequence: –/+n represents the number of base pairs that were deleted/inserted.


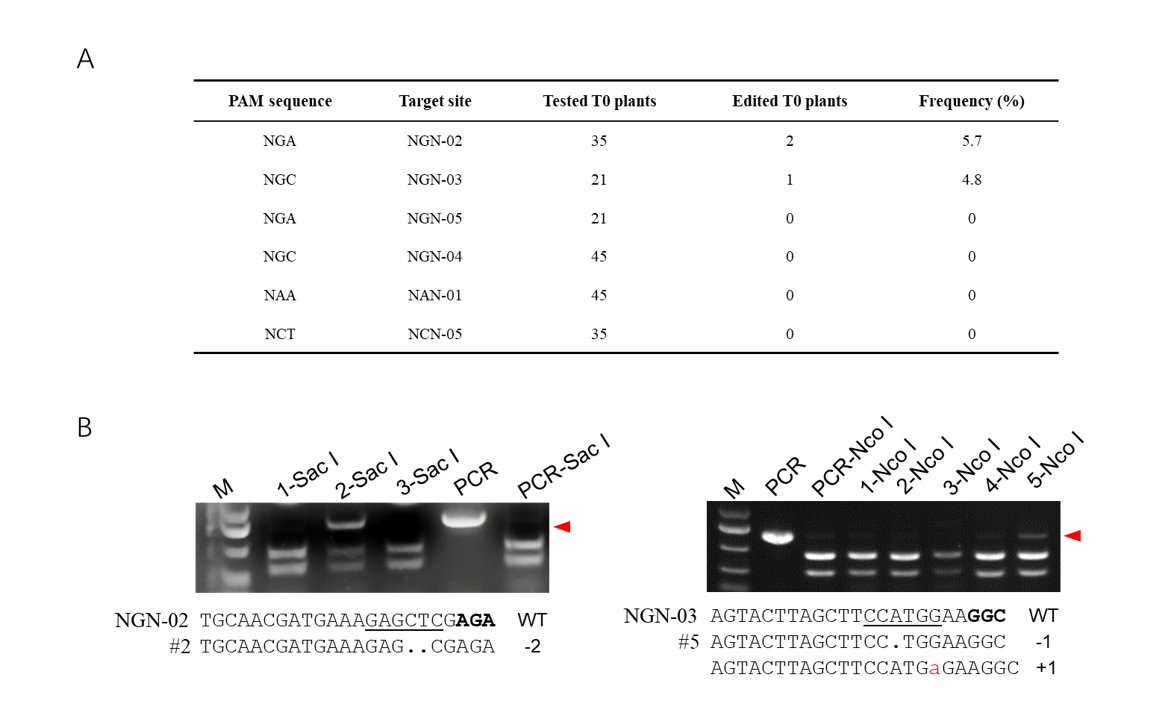


**Supplementary Fig. 5** Mutagenesis induced by SpRY at non-canonical PAMs in T0 plants. **A** SpRY-induced mutagenesis in target sites with NNN PAMs. **B** PCR/RE assays for SpRY-induced mutants in T0 plants. The bands marked by red arrowheads indicate mutations at target sites.

**Supplementary Table 1.** Analysis of targetable PAMs using SpCas9, Cas9-NG, SpG, and SpRY in the genomes of Chinese cabbage and cabbage

| **Species** | **Cas9 variants** | **PAM** | **Genome target** | **Total target PAMs** | **PAM frequencies** |
| --- | --- | --- | --- | --- | --- |
| Chinese cabbage | SpCas9 | NGG | 10,562,473 | 21,140,370 | 11.97% |
|  |  | CCN | 10,577,897 |  |  |
|  | Cas9-NG/SpG | NGN | 52,990,104 | 106,028,983 | 30.03% |
|  |  | NCN | 53,038,879 |  |  |
|  | SpRY | NNN | - | - | ~100% |
| Cabbage | SpCas9 | NGG | 12873675 | 25,753,702 | 10.44% |
|  |  | CCN | 12880027 |  |  |
|  | Cas9-NG/SpG | NGN | 66315078 | 132,685,477 | 26.91% |
|  |  | NCN | 66370399 |  |  |
|  | SpRY | NNN | - | - | ~100% |

**Supplementary Table 2.** The targeted loci and sequences of the targets of the Cas9 variants

| **Nuclease** | **Target name** | | **Gene** | **Target sequences (5’-3’)** | **PAM** |
| --- | --- | --- | --- | --- | --- |
| Cas9-NG | | NGA-01 | *BrPDS1* | CGAGATGCTGACATGGCCAG**AGA** | AGA |
|  |  | NGA-02 | *BrPDS2* | CGAGATGCTGACATGGCCAG**AGA** | AGA |
|  |  | NGA-03 | *BrAOP2* | GAAGAGTAGAACGAGCTCTA**CGA** | CGA |
|  |  | NGT-01 | *BrPDS* | ATGGAGATTGGTATGAAACC**GGT** | GGT |
|  |  | NGT-02 | *BrPDS* | CTCTCGCTTCAAGCAACAGA**GGT** | GGT |
|  |  | NGC-01 | *BrPDS1* | AGTACTTAGCTTCCATGGAA**GGC** | GGC |
|  |  | NGC-02 | *BrPDS2* | AGTACTTAGCTTCCATGGAA**GGC** | GGC |
|  |  | NGC-03 | *BrAOP2* | TCACCATTATGATTGGTACC**AGC** | AGC |
|  |  | NGA-01 | *BoPDS* | TGCAGAGGAGTGGATATCAC**GGA** | GGA |
|  |  | NGA-02 | *BoPDS* | TGCAACGATGAAAGAGCTCG**AGA** | AGA |
|  |  | NGA-03 | *BoPDS1* | CGAGATGCTGACATGGCCAG**AGA** | AGA |
|  |  | NGA-04 | *BoPDS2* | CGAGATGCTGACATGGCCAG**AGA** | AGA |
|  |  | NGA-05 | *BoDMR6* | GTGACCGTCGATCAAGATCT**GGA** | GGA |
|  |  | NGA-06 | *BoDMR6* | AATAGAGGAATTGATATCAG**AGA** | AGA |
|  |  | NGC-01 | *BoPDS1* | AGTACTTAGCTTCCATGGAA**GGC** | GGC |
|  |  | NGC-02 | *BoPDS2* | AGTACTTAGCTTCCATGGAA**GGC** | GGC |
| SpG | | NGA-01 | *BrPDS1* | CGAGATGCTGACATGGCCAG**AGA** | AGA |
|  |  | NGA-02 | *BrPDS2* | CGAGATGCTGACATGGCCAG**AGA** | AGA |
|  |  | NGT-01 | *BrPDS* | ATGGAGATTGGTATGAAACC**GGT** | GGT |
|  |  | NGT-02 | *BrPDS* | CTCTCGCTTCAAGCAACAGA**GGT** | GGT |
|  |  | NGC-01 | *BrPDS1* | AGTACTTAGCTTCCATGGAA**GGC** | GGC |
|  |  | NGC-02 | *BrPDS2* | AGTACTTAGCTTCCATGGAA**GGC** | GGC |
|  |  | NGA-01 | *BoPDS* | TGCAGAGGAGTGGATATCAC**GGA** | GGA |
|  |  | NGA-02 | *BoPDS* | TGCAACGATGAAAGAGCTCG**AGA** | AGA |
|  |  | NGA-03 | *BoPDS1* | CGAGATGCTGACATGGCCAG**AGA** | AGA |
|  |  | NGA-04 | *BoPDS2* | CGAGATGCTGACATGGCCAG**AGA** | AGA |
|  |  | NGA-05 | *BoDMR6* | GTGACCGTCGATCAAGATCT**GGA** | GGA |
|  |  | NGA-06 | *BoDMR6* | AATAGAGGAATTGATATCAG**AGA** | AGA |
|  |  | NGC-01 | *BoPDS1* | AGTACTTAGCTTCCATGGAA**GGC** | GGC |
|  |  | NGC-02 | *BoPDS2* | AGTACTTAGCTTCCATGGAA**GGC** | GGC |
| SpRY | | NAN-01 | *BrPDS* | AGAGAACGGCGCCTTCCATG**GAA** | GAA |
|  |  | NAN-02 | *BrPDS* | TGCAACGATGAAAGAGCTCG**AAA** | AAA |
|  |  | NTN-01 | *BrPDS* | GAAACTGAAGAACACATATG**ATC** | ATC |
|  |  | NCN-01 | *BrAOP2* | CTCTGACACTCTATCATATG**ACA** | ACA |
|  |  | NCN-02 | *BrAOP2* | AACATTGGCATCCTGGATCC**CCA** | CCA |
|  |  | NCN-03 | *BrAOP2* | CTCTGACACTCTATCATATG**ACA** | ACA |
|  |  | NCN-04 | *BrAOP2* | TTGAGTAAATTCGTTAACTT**TCT** | TCT |
|  |  | NGN-01 | *BrPDS1* | CGAGATGCTGACATGGCCAG**AGA** | AGA |
|  |  | NGN-02 | *BrPDS2* | CGAGATGCTGACATGGCCAG**AGA** | AGA |
|  |  | NGN-03 | *BrAOP2* | GAAGAGTAGAACGAGCTCTA**CGA** | CGA |
|  |  | NGN-04 | *BrAOP2* | TCACCATTATGATTGGTACC**AGC** | AGC |
|  |  | NGN-05 | *BrPDS1* | AGTACTTAGCTTCCATGGAA**GGC** | GGC |
|  |  | NGN-06 | *BrPDS2* | AGTACTTAGCTTCCATGGAA**GGC** | GGC |
|  |  | NGN-07 | *BrAOP2* | TAGAGTGTCAGAGGAGCTTA**AGG** | AGG |
|  |  | NAN-01 | *BoPDS* | GAGATGCTGACATGGCCAGA**GAA** | GAA |
|  |  | NTN-01 | *BoDMR6* | CCAAATCGGGCGCAAGCTTG**GTG** | GTG |
|  |  | NTN-02 | *BoDMR6* | CCGAATCGGGAGCAAGCTTG**GTG** | GTG |
|  |  | NCN-01 | *BoDMR6* | TAAACCATAGGTGAGCTCAG**GCT** | GCT |
|  |  | NCN-02 | *BoDMR6* | CATGTCCTGAGCCTGAGCTC**ACT** | ACT |
|  |  | NCN-03 | *BoDMR6* | ATATCTTCCACAGATCGATC**TCG** | TCG |
|  |  | NCN-04 | *BoDMR6* | CATGTCCTGAGCCTGAGCTC**ACC** | ACC |
|  |  | NCN-05 | *BoAOP2* | CTCTGACACTCTATCATATG**ACA** | ACA |
|  |  | NCN-06 | *BoAOP2* | TTGAGTAAATTCGTTAACTT**TCT** | TCT |
|  |  | NCN-07 | *BoAOP2* | ATGGGTTCAGACAGTACTCC**CCA** | CCA |
|  |  | NGN-01 | *BoPDS* | TGCAGAGGAGTGGATATCAC**GGA** | GGA |
|  |  | NGN-02 | *BoPDS* | TGCAACGATGAAAGAGCTCG**AGA** | AGA |
|  |  | NGN-03 | *BoPDS1* | AGTACTTAGCTTCCATGGAA**GGC** | GGC |
|  |  | NGN-04 | *BoPDS2* | AGTACTTAGCTTCCATGGAA**GGC** | GGC |
|  |  | NGN-05 | *BoPDS1* | CGAGATGCTGACATGGCCAG**AGA** | AGA |
|  |  | NGN-06 | *BoPDS2* | CGAGATGCTGACATGGCCAG**AGA** | AGA |
|  |  | NGN-07 | *BoDMR6* | GTGACCGTCGATCAAGATCT**GGA** | GGA |
|  |  | NGN-08 | *BoDMR6* | AATAGAGGAATTGATATCAG**AGA** | AGA |
|  |  | NGN-09 | *BoAOP2.1* | TAGAGTGTCAGAGGAGCTTA**AGG** | AGG |
|  |  | NGN-10 | *BoAOP2.2* | TAGAGTGTCAGAGGAGCTTA**AGG** | AGG |
| SpRYn-ABE8e | | ABE8e-01 | *BrPDS* | CATGGCCAGAGAAAATAAAG**TTT** | TTT |
|  |  | ABE8e-02 | *BrPDS* | CTTCCATGGAAGGCGCCGTT**CTC** | CTC |

**Supplementary Table 3.** Targeted mutagenesis in T-DNA induced by Cas9-NG, SpG, and SpRY

| **Nuclease** | **Target name** | **Gene** | **Target sequences (5’-3’)** | **Self-editing frequency (%)** |  |
| --- | --- | --- | --- | --- | --- |
| Cas9-NG | NGA-03 | *BoPDS1* | CGAGATGCTGACATGGCCAG**AGA** | 0 |  |
|  | NGC-03 | *BrAOP2* | TCACCATTATGATTGGTACC**AGC** | 5.21 |  |
|  | NGC-02 | *BoPDS2* | AGTACTTAGCTTCCATGGAA**GGC** | 0 |  |
|  | NGA-03 | *BrAOP2* | GAAGAGTAGAACGAGCTCTA**CGA** | 0 |  |
| SpG | NGA-03 | *BoPDS1* | CGAGATGCTGACATGGCCAG**AGA** | 0 |  |
|  | NGA-02 | *BoPDS* | TGCAACGATGAAAGAGCTCG**AGA** | 3.47 |  |
|  | NGC-02 | *BoPDS2* | AGTACTTAGCTTCCATGGAA**GGC** | 0 |  |
| SPRY | NCN-01 | *BrAOP2* | CTCTGACACTCTATCATATG**ACA** | 45.41 | |
|  | NGN-02 | *BoPDS* | TGCAACGATGAAAGAGCTCG**AGA** | 13.89 |  |
|  | NCN-02 | *BrAOP2* | AACATTGGCATCCTGGATCC**CCA** | 5.26 |  |
|  | NGN-04 | *BoPDS2* | AGTACTTAGCTTCCATGGAA**GGC** | 0 |  |
|  | NGN-05 | *BoPDS1* | CGAGATGCTGACATGGCCAG**AGA** | 0 |  |
|  | NGN-04 | *BrAOP2* | TCACCATTATGATTGGTACC**AGC** | 10.42 |  |
|  | NGN-03 | *BrAOP2* | GAAGAGTAGAACGAGCTCTA**CGA** | 0 |  |

Each PAM motif is shown in bold.

**Supplementary Table 4.** The primers used in this study

| **Primer name** | **Primer sequence** | **Experiment** |
| --- | --- | --- |
| pB-XbaI-F1 | CTCGAGTAATCTAGAATGGCCCCAAAGAAGAAGCG | Constructing the vectors pBSE-SpG, SpRY, SpRYn-ABE8e |
| pB-SacI-R1 | GAAAGCTCTGAGCTCTCACACCTTGCGCTTCTTCTTC |  |
| PDS1-F | TCGAATTACCCGAACTATCC | PCR amplification |
| PDS1-R | GAGATATTGTGTGATCCATCATC |  |
| 8e1-F | AGGATGAAGATGGAGATTGG |  |
| 8e1-R | TGACCTCCGACCATAGCC |  |
| 8e2-F | ATCCTAACCGGTCAATGCT |  |
| 8e2-R | TGATAGAGTCGTCTCCGACA |  |
| PDSNcoI-GAA-F | CAGAAGTAACCTTCTGAGTGTG | Amplifying the sequence |
| PDSNdeI-ATC-F | ATGCAATGCATTTTGATAGC |  |
| PDSNdeI-ATC-R | CACACTCAGAAGGTTACTTCTG |  |
| PDSMscI-AGA-F | AACTTATTTGGAGAACTTGGG |  |
| PDSMscI-AGA-R | TATCAAAATGCATTGCATTG |  |
| PDSPstI-CGA-F | AGGATGAAGATGGAGATTGG |  |
| PDSPstI-CGA-R | TGACCTCCGACCATAGCC |  |
| AOP2-ACA-F | ATCTCTCGGACCAAACCC |  |
| AOP2-ACA-R | ATCAGGGCG TAGCAGTTG |  |
| AOP2-CGA-F | CAATAGCATTGTTCTCGACTC |  |
| AOP2-CGA-R | CAATTCGAAAACGCTATCC |  |
| AOP2-AGC-F | GGATGTAATGGTGAGAAGAATG |  |
| AOP2-AGC-R | CTCTATGAAACGGACGAGAC |  |
| AOP2-CCA-R | CCTGGTACTCTTGTTACCCTC |  |
| 5BrPDS1-F | ATCCTAACCGGTCAATGCT |  |
| 5BrPDS1-R | TGATAGAGTCGTCTCCGACA |  |
| 6BrPDS1-F | ACTTGCTCTTTAGCAGGTGA |  |
| 6BrPDS1-R | TCATGTTGATACAGTTGTCTCC |  |
| Bo3.2-BrPDS2-F | AGCTAGTTGGTGTGCCAGTT |  |
| Bo3.2-BrPDS2-R | TGATAGAGTCGTCTCCGACA |  |
| 7BrPDS1-F | AACTTATTTGGAGAACTTGGGA |  |
| 7BrPDS1-R | CTATCAAAATGCATTGCATTG |  |
| 7BrPDS2-F | TGAGATTAACTAATGGTCAACAA |  |
| 7BrPDS2-R | GATCCATGTTTCTCCTGTCA |  |
| BrAOP2.1F | ATGGGTGCAGACACTCCTCA |  |
| BrAOP2.1R | CTGGATCCCTAGACTCTCAGAG |  |
| BrAOP2.2F | ATGGGTTCAGACAGTACTCCTC |  |
| BrAOP2.2R | CGGAAATACCATTATGAGTCAT |  |
| BrAOP2.3F | ATGGGTTCAGACAGTACTCCC |  |
| BrAOP2.3R | GGAAAGACCATTATGAGTCGAG |  |
| 1-PDS1-NGS-F | TGCATGGATAGTGCTGAATTGTCCTCTGT |  |
| 1-PDS1-NGS-R | TCCAATTTAGGAAGAAACAAAGAACCACA |  |
| 1-PDS2-NGS-F | ATTGTAGCTGTTAGGACAACACATGTTGG |  |
| 1-PDS2-NGS-R | TGCACCTTGGAAGCAACAAAGAAACTCTAT |  |
| 2-PDS2-NGS-F | GCGGATTTTGAGATTAACTAATGGTCAACAA |  |
| 2-PDS2-NGS-R | GAAGGGAAGGACATCTGGGAAATCAAAT |  |
| 3-PDS1-NGS-F | AATTGTATTAGTGCTGAATTGTCCTCTGT |  |
| 3-PDS1-NGS-R | CTTGGTAAAGGAAGAAACAAAGAACCACA |  |
| 3-PDS2-NGS-F | TGCTCCTATGTTAGGACAACACATGTTGG |  |
| 3-PDS2-NGS-R | GACGTTCTGGAAGCAACAAAGAAACTCTAT |  |
| 4-PDS2-NGS-F | GTAGAAGATGAGATTAACTAATGGTCAACAA |  |
| 4-PDS2-NGS-R | GGAACCCAGGACATCTGGGAAATCAAAT |  |
| 5-NGS-F | GCTAGCAAGGAGATTACATCATCGAACCA |  |
| 5-NGS-R | CGGGCTCGGCAGTTCTTGATATTAGTTGAACC |  |
| 6-NGS-F | CAATTAGTCTAAAGATTCTGATGATGGTGC |  |
| 6-NGS-R | TTTATGCTCAGCACCAACACTAGATTTAGC |  |
| 7-NGS-F | TGAGGCCAGGAGATTACATCATCGAACCA |  |
| 7-NGS-R | GAGGTTACGCAGTTCTTGATATTAGTTGAACC |  |
| 8-NGS-F | CTGGGACGCTAAAGATTCTGATGATGGTGC |  |
| 8-NGS-R | TTATTCGGCAGCACCAACACTAGATTTAGC |  |
| 33-AOP2.1-NGS-F | CAGGAGTGTATCTCTCGGACCAAACCC |  |
| 33-AOP2.1-NGS-R | TACGAGTGCTCAGAGAGACCATTATGAGTTG |  |
| 33-AOP2.2-NGS-F | AGTGGAAGAACCAGGAAGTGAGAAGTGG |  |
| 33-AOP2.2-NGS-R | ACAATTCGTCCTGGATCCCCAGACTCT |  |
| 33-AOP2.3-NGS-F | TTGAGGCCATGGGTTCAGACAGTACTCCC |  |
| 33-AOP2.3-NGS-R | ATACTCATGAGACACGTTCCTCCGTTTA |  |
| 34-AOP2.2-NGS-F | AAGAAGAAAGTGTCAGAGGAGCTTAAGGA |  |
| 34-AOP2.2-NGS-R | CTTCGATTTCATAACAATACGGACCTGG |  |
| 35-PDS1-NGS-F | AAATTTTAGTGTTGCAGAAGTAACCTTCTG |  |
| 35-PDS1-NGS-R | AGGGGAGGACTAACCTTGGAGTTTTGACG |  |
| 36BrPDS1NGS-F | ACCTCAAACTATCAGGTCCGTGTACAAGAC |  |
| 36BrPDS1NGS-R | AATCAGCACAGGGTGACTGGTTAACAAATA |  |
| 36BrPDS2NGS-F | GCAAGTTGCAGGTCTGTGTACAAGACGATC |  |
| 36BrPDS2NGS-R | TATTTGAAAATAAACCGGATCATTTACCAG |  |
| 37BrPDS1NGS-F | GGTACGGTTAGTGCTGAATTGTCCTCTGT |  |
| 37BrPDS1NGS-R | GGGTGAGTAGGAAGAAACAAAGAACCACA |  |
| 37BrPDS2NGS-F | CTATTTCTTCAAGTTGTTTGAACCTTTCAA |  |
| 37BrPDS2NGS-R | CATGTATAGCTTTCTCATCCATTGTTCAA |  |
| 38BrPDS1NGS-F | GAGATATCCTATCAGGTCCGTGTACAAGAC |  |
| 38BrPDS1NGS-R | ATGTGGTCCAGGGTGACTGGTTAACAAATA |  |
| 38BrPDS2NGS-F | AAGGTAATCAGGTCTGTGTACAAGACGATC |  |
| 38BrPDS2NGS-R | CATGAGGGAATAAACCGGATCATTTACCAG |  |
| 39BrPDS1NGS-F | GTACGGCGTAGTGCTGAATTGTCCTCTGT |  |
| 39BrPDS1NGS-R | AGAGTGTTAGGAAGAAACAAAGAACCACA |  |
| 39BrPDS2NGS-F | CTAGCGCGTCAAGTTGTTTGAACCTTTCAA |  |
| 39BrPDS2NGS-R | AGATGCCCGCTTTCTCATCCATTGTTCAA |  |
| 310BrPDS1-NGS-F | TCCGGGCTCTATCAGGTCCGTGTACAAGAC |  |
| 310BrPDS1-NGS-R | GATTGGACCAGGGTGACTGGTTAACAAATA |  |
| 310BrPDS2-NGS-F | TTTATCCCCAGGTCTGTGTACAAGACGATC |  |
| 310BrPDS2-NGS-R | GGTCTCAAAATAAACCGGATCATTTACCAG |  |
| 311BrPDS1-NGS-F | GCGGGAGCTAGTGCTGAATTGTCCTCTGT |  |
| 311BrPDS1-NGS-R | CGCCGCCAAGGAAGAAACAAAGAACCACA |  |
| 311BrPDS2-NGS-F | TCCCGGTTTCAAGTTGTTTGAACCTTTCAA |  |
| 311BrPDS2-NGS-R | CCAATATCGCTTTCTCATCCATTGTTCAA |  |
| 312BrAOP2.1-NGS-F | AACTGCAATATCTCTCGGACCAAACCC |  |
| 312BrAOP2.1-NGS-R | CGGTTAGGCTCAGAGAGACCATTATGAGTTG |  |
| 312BrAOP2.2-NGS-F | TACACATAAACCAGGAAGTGAGAAGTGG |  |
| 312BrAOP2.2-NGS-R | AAAGGGCTTCCTGGATCCCCAGACTCT |  |
| 312BrAOP2.3-NGS-F | TATCTGGGATGGGTTCAGACAGTACTCCC |  |
| 312BrAOP2.3-NGS-R | AAATCTGTGAGACACGTTCCTCCGTTTA |  |
| 313BrAOP2.1-NGS-F | GAAGCTGGGGTGTCATATGATAGAGTGTCAG |  |
| 313BrAOP2.1-NGS-R | TTCTACATTTGACAAGACGTTACCTCATACT |  |
| 313BrAOP2.2-NGS-F | CATTGTTGAGTGTCAGAGGAGCTTAAGGA |  |
| 313BrAOP2.2-NGS-R | ATTGCGGCTCATAACAATACGGACCTGG |  |
| 313BrAOP2.3-NGS-F | GCCATTCTGAGTGTCAGAGGAGCTAAAGC |  |
| 313BrAOP2.3-NGS-R | AGTACGGTGCTGTTCACATAACATAACCTT |  |
| Bo1.1-PDS1-F | TCGAATTACCCGAACTATCC |  |
| Bo1.1-PDS1-R | GAGATATTGTGTGATCCATCATC |  |
| Bo4.1-PDS1-F | GTGTATGCTGACATGTCGTTAA |  |
| Bo4.1-PDS1-R | ATGTTGATAGAGTCGCCTCC |  |
| Bo4.1-PDS2-F | GAGAAACTCTTCCCTGACGA |  |
| Bo4.1-PDS2-R | TCATGTTGATACAGTTGTCTCC |  |
| Bo4.2-PDS1-F | GGTGAAAGTTGGTGCATATC |  |
| Bo4.2-PDS1-R | CCTGAAGAAACCGATTCAA |  |
| Bo4.2-PDS2-F | TATTCTTGCTGGAAGTCGG |  |
| Bo4.2-PDS2-R | AACACCTCATCAGTCACGC |  |
| Bo7.1-DMR6.1-F | CAGGCATACAGATTTCTATCGT |  |
| Bo7.1-DMR6.1-R | GGGCGGAAACTCATAATAA |  |
| Bo7.2-DMR6.1-F | CCAAAATCATGTAGTTTCGTG |  |
| Bo7.2-DMR6.1-R | GGGAATAGTGAGTTTAAGGACTC |  |
| Bo9.1- DMR6.1-R | CTGAAGTCCAAAGCTATGGC |  |
| Bo8.1-DMR6.2-F | GCTTAAACCCACTTCATACAAT |  |
| Bo8.1-DMR6.2-R | GACCGACGGTTAGTGGAAT |  |
| Bo8.2-DMR6.2-F | CATCATGAGCTTCAATGCA |  |
| Bo8.2-DMR6.2-R | TAAGCAAGGTTGGTCCAAG |  |
| Bo12.1-DMR6.1-F | TTGTTATTATTCACAGCGGTG |  |
| Bo13.1-AOP2.1-F | TGTTGTGGGTGATTAAAGAGTT |  |
| Bo13.1-AOP2.1-R | ATGCGTGAAACGTGAGATT |  |
| Bo13.1-AOP2.2-F | AGCGAAGATCCCTTAAGCA |  |
| Bo13.1-AOP2.2-R | AACAGGGGTTTACGAACAAG |  |
| Bo13.1-AOP2.3-F | AACCAACTTGGTCTCCTAGAAT |  |
| Bo13.1-AOP2.3-R | AAGGGTTTAGGGTTTGGAGT |  |
| Bo14.2-AOP2.3-R | TCCAGAAATACCCAAAGATATC |  |
| 316BoPDS1-NGS-F | TTAGGATGGTTGTCTCTGTGTTGCAGAAG |  |
| 316BoPDS1-NGS-R | AGCTCCACGACGACATGGTACTTGAGAATC |  |
| 317BoPDS1-NGS-F | CTTAGTCCGTTGTCTCTGTGTTGCAGAAG |  |
| 317BoPDS1-NGS-R | TCTGGTAGGACGACATGGTACTTGAGAATC |  |
| 318BoPDS1-NGS-F | TATTGGTAGTTGTCTCTGTGTTGCAGAAG |  |
| 318BoPDS1-NGS-R | TCGTAAGTGACGACATGGTACTTGAGAATC |  |
| 319BoPDS1-NGS-F | GACTCACTGTTGTCTCTGTGTTGCAGAAG |  |
| 319BoPDS1-NGS-R | AGGTATTCGACGACATGGTACTTGAGAATC |  |
| 13-PDS1-NGS-F | TAAGACGTGTTGTCTCTGTGTTGCAGAAG |  |
| 13-PDS1-NGS-R | GAAGTAGCGACGACATGGTACTTGAGAATC |  |
| 14-PDS1-NGS-F | ATTGGTGAGTTGTCTCTGTGTTGCAGAAG |  |
| 14-PDS1-NGS-R | TTGGGGACGACGACATGGTACTTGAGAATC |  |
| 15-PDS1-NGS-F | GGAAGTGTTATCAGGTCTGTGTACAAGACG |  |
| 15-PDS1-NGS-R | TGACACCAAACCGGATCATTTACCAGAA |  |
| 15-PDS2-NGS-F | CTTGGGTATACAAGACCATCCCAGACTG |  |
| 15-PDS2-NGS-R | ATAATCAACTGGTTAAAATCCAGGATGG |  |
| 16-PDS1-NGS-F | CATCGACAGCATAGATTTGATTTGTATTGGT |  |
| 16-PDS1-NGS-R | GTGAATATAAGGAAGCAACAAAGAAACTC |  |
| 16-PDS2-NGS-F | GGAATGCTGTTGTAGCGCATAGTGCTGA |  |
| 16-PDS2-NGS-R | AATTACTGCAGCTACCAATCAAACAAACC |  |
| 17-PDS1-NGS-F | TGGATAACTATCAGGTCTGTGTACAAGACG |  |
| 17-PDS1-NGS-R | CTCAAGGCAACCGGATCATTTACCAGAA |  |
| 17-PDS2-NGS-F | AGTCTCTGTACAAGACCATCCCAGACTG |  |
| 17-PDS2-NGS-R | TCTTGATACTGGTTAAAATCCAGGATGG |  |
| 18-PDS1-NGS-F | TATCTATCGCATAGATTTGATTTGTATTGGT |  |
| 18-PDS1-NGS-R | GTATTCATAAGGAAGCAACAAAGAAACTC |  |
| 18-PDS2-NGS-F | CGTAATAAGTTGTAGCGCATAGTGCTGA |  |
| 18-PDS2-NGS-R | CACTTGATCAGCTACCAATCAAACAAACC |  |
| 19-PDS1-NGS-F | AATAGGGTTATCAGGTCTGTGTACAAGACG |  |
| 19-PDS1-NGS-R | TGCCAAAAAACCGGATCATTTACCAGAA |  |
| 19-PDS2-NGS-F | AGGGGCGGTACAAGACCATCCCAGACTG |  |
| 19-PDS2-NGS-R | ATCTTGATCTGGTTAAAATCCAGGATGG |  |
| 20-PDS1-NGS-F | CTCCATAGGCATAGATTTGATTTGTATTGGT |  |
| 20-PDS1-NGS-R | AGGGGGGGAAGGAAGCAACAAAGAAACTC |  |
| 320BoDMR6.1-NGS-F | AATTGCTAGAAGATTTCCCTCTCATCGA |  |
| 320BoDMR6.1-NGS-R | CTCTTGATAATAGAAACAAAGGCTAGTGCA |  |
| 321BoDMR6.1-NGS-F | CAGAATCAAGAGGAATTGATATCAGAGAGCT |  |
| 321BoDMR6.1-NGS-R | AGAAGGGTTTGATGACAAAAGCATCAGG |  |
| 322BoDMR6.2-NGS-F | GGTTAACAACTCTCCAACCGTCCATGT |  |
| 322BoDMR6.2-NGS-R | GAATAAATCCTAATCCTTTTCAGGCACA |  |
| 323BoDMR6.2-NGS-F | TAATAGATGCAGGGAAGTTGTGAGTAGAT |  |
| 323BoDMR6.2-NGS-R | ATTTTTGGTGTAGACCACAAACGGTAGC |  |
| 324BoDMR6.1-NGS-F | GACGGAATAGAGGAATTGATATCAGAGAGCT |  |
| 324BoDMR6.1-NGS-R | TATTCGAGTTGATGACAAAAGCATCAGG |  |
| 325BoDMR6.1-NGS-F | TTTTACAACAAAATCATGTAGTTTCGTGG |  |
| 325BoDMR6.1-NGS-R | CTAAGGGAAGCACTTTCTTCATGTAATCTTT |  |
| 326BoDMR6.1-NGS-F | TTATCTACAGAGGAATTGATATCAGAGAGCT |  |
| 326BoDMR6.1-NGS-R | TGAGCGAATTGATGACAAAAGCATCAGG |  |
| 327BoDMR6.1-NGS-F | CGGAATCACAAAATCATGTAGTTTCGTGG |  |
| 327BoDMR6.1-NGS-R | GAAGACACAGCACTTTCTTCATGTAATCTTT |  |
| 328BoDMR6.1-NGS-F | TTCAGTGCAGAGGAATTGATATCAGAGAGCT |  |
| 328BoDMR6.1-NGS-R | AATGGCCGTTGATGACAAAAGCATCAGG |  |
| 329BoDMR6.1-NGS-F | CCGGTAAGCAAAATCATGTAGTTTCGTGG |  |
| 329BoDMR6.1-NGS-R | GGCTTGTTAGCACTTTCTTCATGTAATCTTT |  |
| 330BoDMR6.1-NGS-F | TACAGGGACAAAGCTTTTATCCACCGG |  |
| 330BoDMR6.1-NGS-R | TGGGAGCTTTCATATACCTTATCAAAGCGTG |  |
| 330BoDMR6.2-NGS-F | ATACAATCACTCTCCAACCGTCCATGT |  |
| 330BoDMR6.2-NGS-R | CCTTGTTCCCTAATCCTTTTCAGGCACA |  |
| 21-AOP2.1-NGS-F | CGTTATTTAGACACTCCTCAACTTCCAGT |  |
| 21-AOP2.1-NGS-R | TACAAAGAAATATCCAGTGTACGGTTTCG |  |
| 21-AOP2.2-NGS-F | AAACGTGTGACAGTACTCCCCAACTTCC |  |
| 21-AOP2.2-NGS-R | TTTGTGTTCCAGTGTAGGGTTTTGGAGA |  |
| 21-AOP2.3-NGS-F | GTCTTGGGATGGGTTCAGACAGTACTCCT |  |
| 21-AOP2.3-NGS-R | GGGTGCTTTTGGGAGACACGTTTCTTCT |  |
| 22-AOP2.1-NGS-F | GAAAACGAGCTAAACAGAGAAACGTGTCTC |  |
| 22-AOP2.1-NGS-R | AAAAGCCTTTGATGAAAGATTAACAGGAATT |  |
| 22-AOP2.2-NGS-F | TGTCGGGTTTACCAGTTGAGGCTAAACG |  |
| 22-AOP2.2-NGS-R | TTTTCTGAACATAATTACCGCTTAGCGAT |  |
| 22-AOP2.3-NGS-F | TCTTTGTGGGAAACCATGAAAGAGCTTT |  |
| 22-AOP2.3-NGS-R | ATAACGCTCTTTATGAAGTAATATTGGGGTT |  |
| 23-AOP2.2-NGS-F | TTTCGCATCGAAGATCCCTTAAGCAAAG |  |
| 23-AOP2.2-NGS-R | GCAGTCTATTCCTGGTTTAGGGTTTGGT |  |
| 23-AOP2.3-NGS-F | CGTTCTCATGAATGCTTTCATTTGTTGTT |  |
| 23-AOP2.3-NGS-R | AGGTTTTAAAGCGCCGTAGTCTTCAAG |  |
| 24-AOP2.1-NGS-F | GGGTATACGACACTCCTCAACTTCCAGTC |  |
| 24-AOP2.1-NGS-R | GGGTTGAGTATCCAGTGTACGGTTTCGG |  |
| 24-AOP2.3-NGS-F | GCGGGGAGGGGTTCAGACAGTACTCCTCA |  |
| 24-AOP2.3-NGS-R | ATGGCCATGTTTGGGAGACACGTTTCTT |  |
| BUN-F | GTTGTAAAACGACGGCCAG | Self-editing detection |
| BUN-R | CAAGCTTATTGGTTTATCTCATCGG |  |
| 3Z-F | CTCCGAGCGGCCCATTTAAGTTGAAAAC |  |
| 3Z-R | AAGCAACCAGAAATTGAACGCCGAAGAA |  |

**Supplementary Table 5.** The complete nucleotide sequences of Cas9-NG, SpG, SpRY and SpRYn-ABE8e

| Cas9-NG | ATGGCTCCGAAGAAGAAGAGGAAGGTTGGCATCCACGGGGTGCCAGCTGCTGACAAGAAGTACTCGATCGGCCTCGATATTGGGACTAACTCTGTTGGCTGGGCCGTGATCACCGACGAGTACAAGGTGCCCTCAAAGAAGTTCAAGGTCCTGGGCAACACCGATCGGCATTCCATCAAGAAGAATCTCATTGGCGCTCTCCTGTTCGACAGCGGCGAGACGGCTGAGGCTACGCGGCTCAAGCGCACCGCCCGCAGGCGGTACACGCGCAGGAAGAATCGCATCTGCTACCTGCAGGAGATTTTCTCCAACGAGATGGCGAAGGTTGACGATTCTTTCTTCCACAGGCTGGAGGAGTCATTCCTCGTGGAGGAGGATAAGAAGCACGAGCGGCATCCAATCTTCGGCAACATTGTCGACGAGGTTGCCTACCACGAGAAGTACCCTACGATCTACCATCTGCGGAAGAAGCTCGTGGACTCCACAGATAAGGCGGACCTCCGCCTGATCTACCTCGCTCTGGCCCACATGATTAAGTTCAGGGGCCATTTCCTGATCGAGGGGGATCTCAACCCGGACAATAGCGATGTTGACAAGCTGTTCATCCAGCTCGTGCAGACGTACAACCAGCTCTTCGAGGAGAACCCCATTAATGCGTCAGGCGTCGACGCGAAGGCTATCCTGTCCGCTAGGCTCTCGAAGTCTCGGCGCCTCGAGAACCTGATCGCCCAGCTGCCGGGCGAGAAGAAGAACGGCCTGTTCGGGAATCTCATTGCGCTCAGCCTGGGGCTCACGCCCAACTTCAAGTCGAATTTCGATCTCGCTGAGGACGCCAAGCTGCAGCTCTCCAAGGACACATACGACGATGACCTGGATAACCTCCTGGCCCAGATCGGCGATCAGTACGCGGACCTGTTCCTCGCTGCCAAGAATCTGTCGGACGCCATCCTCCTGTCTGATATTCTCAGGGTGAACACCGAGATTACGAAGGCTCCGCTCTCAGCCTCCATGATCAAGCGCTACGACGAGCACCATCAGGATCTGACCCTCCTGAAGGCGCTGGTCAGGCAGCAGCTCCCCGAGAAGTACAAGGAGATCTTCTTCGATCAGTCGAAGAACGGCTACGCTGGGTACATTGACGGCGGGGCCTCTCAGGAGGAGTTCTACAAGTTCATCAAGCCGATTCTGGAGAAGATGGACGGCACGGAGGAGCTGCTGGTGAAGCTCAATCGCGAGGACCTCCTGAGGAAGCAGCGGACATTCGATAACGGCAGCATCCCACACCAGATTCATCTCGGGGAGCTGCACGCTATCCTGAGGAGGCAGGAGGACTTCTACCCTTTCCTCAAGGATAACCGCGAGAAGATCGAGAAGATTCTGACTTTCAGGATCCCGTACTACGTCGGCCCACTCGCTAGGGGCAACTCCCGCTTCGCTTGGATGACCCGCAAGTCAGAGGAGACGATCACGCCGTGGAACTTCGAGGAGGTGGTCGACAAGGGCGCTAGCGCTCAGTCGTTCATCGAGAGGATGACGAATTTCGACAAGAACCTGCCAAATGAGAAGGTGCTCCCTAAGCACTCGCTCCTGTACGAGTACTTCACAGTCTACAACGAGCTGACTAAGGTGAAGTATGTGACCGAGGGCATGAGGAAGCCGGCTTTCCTGTCTGGGGAGCAGAAGAAGGCCATCGTGGACCTCCTGTTCAAGACCAACCGGAAGGTCACGGTTAAGCAGCTCAAGGAGGACTACTTCAAGAAGATTGAGTGCTTCGATTCGGTCGAGATCTCTGGCGTTGAGGACCGCTTCAACGCCTCCCTGGGGACCTACCACGATCTCCTGAAGATCATTAAGGATAAGGACTTCCTGGACAACGAGGAGAATGAGGATATCCTCGAGGACATTGTGCTGACACTCACTCTGTTCGAGGACCGGGAGATGATCGAGGAGCGCCTGAAGACTTACGCCCATCTCTTCGATGACAAGGTCATGAAGCAGCTCAAGAGGAGGAGGTACACCGGCTGGGGGAGGCTGAGCAGGAAGCTCATCAACGGCATTCGGGACAAGCAGTCCGGGAAGACGATCCTCGACTTCCTGAAGAGCGATGGCTTCGCGAACCGCAATTTCATGCAGCTGATTCACGATGACAGCCTCACATTCAAGGAGGATATCCAGAAGGCTCAGGTGAGCGGCCAGGGGGACTCGCTGCACGAGCATATCGCGAACCTCGCTGGCTCGCCAGCTATCAAGAAGGGGATTCTGCAGACCGTGAAGGTTGTGGACGAGCTGGTGAAGGTCATGGGCAGGCACAAGCCTGAGAACATCGTCATTGAGATGGCCCGGGAGAATCAGACCACGCAGAAGGGCCAGAAGAACTCACGCGAGAGGATGAAGAGGATCGAGGAGGGCATTAAGGAGCTGGGGTCCCAGATCCTCAAGGAGCACCCGGTGGAGAACACGCAGCTGCAGAATGAGAAGCTCTACCTGTACTACCTCCAGAATGGCCGCGATATGTATGTGGACCAGGAGCTGGATATTAACAGGCTCAGCGATTACGACGTCGATCATATCGTTCCACAGTCATTCCTGAAGGATGACTCCATTGACAACAAGGTCCTCACCAGGTCGGACAAGAACCGGGGCAAGTCTGATAATGTTCCTTCAGAGGAGGTCGTTAAGAAGATGAAGAACTACTGGCGCCAGCTCCTGAATGCCAAGCTGATCACGCAGCGGAAGTTCGATAACCTCACAAAGGCTGAGAGGGGCGGGCTCTCTGAGCTGGACAAGGCGGGCTTCATCAAGAGGCAGCTGGTCGAGACACGGCAGATCACTAAGCACGTTGCGCAGATTCTCGACTCACGGATGAACACTAAGTACGATGAGAATGACAAGCTGATCCGCGAGGTGAAGGTCATCACCCTGAAGTCAAAGCTCGTCTCCGACTTCAGGAAGGATTTCCAGTTCTACAAGGTTCGGGAGATCAACAATTACCACCATGCCCATGACGCGTACCTGAACGCGGTGGTCGGCACAGCTCTGATCAAGAAGTACCCAAAGCTCGAGAGCGAGTTCGTGTACGGGGACTACAAGGTTTACGATGTGAGGAAGATGATCGCCAAGTCGGAGCAGGAGATTGGCAAGGCTACCGCCAAGTACTTCTTCTACTCTAACATTATGAATTTCTTCAAGACAGAGATCACTCTGGCCAATGGCGAGATCCGGAAGCGCCCCCTCATCGAGACGAACGGCGAGACGGGGGAGATCGTGTGGGACAAGGGCAGGGATTTCGCGACCGTCAGGAAGGTTCTCTCCATGCCACAAGTGAATATCGTCAAGAAGACAGAGGTCCAGACTGGCGGGTTCTCTAAGGAGTCAATTAGACCTAAGCGGAACAGCGACAAGCTCATCGCCCGCAAGAAGGACTGGGATCCGAAGAAGTACGGCGGGTTCGTTAGCCCCACTGTGGCCTACTCGGTCCTGGTTGTGGCGAAGGTTGAGAAGGGCAAGTCCAAGAAGCTCAAGAGCGTGAAGGAGCTGCTGGGGATCACGATTATGGAGCGCTCCAGCTTCGAGAAGAACCCGATCGATTTCCTGGAGGCGAAGGGCTACAAGGAGGTGAAGAAGGACCTGATCATTAAGCTCCCCAAGTACTCACTCTTCGAGCTGGAGAACGGCAGGAAGCGGATGCTGGCTTCCGCTAGATTTCTGCAGAAGGGGAACGAGCTGGCTCTGCCGTCCAAGTATGTGAACTTCCTCTACCTGGCCTCCCACTACGAGAAGCTCAAGGGCAGCCCCGAGGACAACGAGCAGAAGCAGCTGTTCGTCGAGCAGCACAAGCATTACCTCGACGAGATCATTGAGCAGATTTCCGAGTTCTCCAAGCGCGTGATCCTGGCCGACGCGAATCTGGATAAGGTCCTCTCCGCGTACAACAAGCACCGCGACAAGCCAATCAGGGAGCAGGCTGAGAATATCATTCATCTCTTCACCCTGACGAACCTCGGCGCCCCTAGAGCTTTCAAGTACTTCGACACAACTATCGATCGCAAGGTTTACAGAAGCACTAAGGAGGTCCTGGACGCGACCCTCATCCACCAGTCGATTACCGGCCTCTACGAGACGCGCATCGACCTGTCTCAGCTCGGGGGCGACAAGCGGCCAGCGGCGACGAAGAAGGCGGGGCAGGCGAAGAAGAAGAAGTGA |
| --- | --- |
| SpG | ATGGCCCCAAAGAAGAAGCGCAAGGTCGACAAGAAGTACTCCATCGGCCTCGACATCGGCACCAATTCTGTTGGCTGGGCCGTGATCACCGACGAGTACAAGGTGCCGTCCAAGAAGTTCAAGGTCCTCGGCAACACCGACCGCCACTCCATCAAGAAGAATCTCATCGGCGCCCTGCTGTTCGACTCTGGCGAGACAGCCGAGGCTACAAGGCTCAAGAGGACCGCTAGACGCAGGTACACCAGGCGCAAGAACCGCATCTGCTACCTCCAAGAGATCTTCTCCAACGAGATGGCCAAGGTGGACGACAGCTTCTTCCACAGGCTCGAGGAGAGCTTCCTCGTCGAGGAGGACAAGAAGCACGAGCGCCATCCGATCTTCGGCAACATCGTGGATGAGGTGGCCTACCACGAGAAGTACCCGACCATCTACCACCTCCGCAAGAAGCTCGTCGACTCCACCGATAAGGCCGACCTCAGGCTCATCTACCTCGCCCTCGCCCACATGATCAAGTTCAGGGGCCACTTCCTCATCGAGGGCGACCTCAACCCGGACAACTCCGATGTGGACAAGCTGTTCATCCAGCTCGTGCAGACCTACAACCAGCTGTTCGAGGAGAACCCGATCAACGCCTCTGGCGTTGACGCCAAGGCTATTCTCTCTGCCAGGCTCTCTAAGTCCCGCAGGCTCGAGAATCTGATCGCCCAACTTCCGGGCGAGAAGAAGAATGGCCTCTTCGGCAACCTGATCGCCCTCTCTCTTGGCCTCACCCCGAACTTCAAGTCCAACTTCGACCTCGCCGAGGACGCCAAGCTCCAGCTTTCCAAGGACACCTACGACGACGACCTCGACAATCTCCTCGCCCAGATTGGCGATCAGTACGCCGATCTGTTCCTCGCCGCCAAGAATCTCTCCGACGCCATCCTCCTCAGCGACATCCTCAGGGTGAACACCGAGATCACCAAGGCCCCACTCTCCGCCTCCATGATCAAGAGGTACGACGAGCACCACCAGGACCTCACACTCCTCAAGGCCCTCGTGAGACAGCAGCTCCCAGAGAAGTACAAGGAGATCTTCTTCGACCAGTCCAAGAACGGCTACGCCGGCTACATCGATGGCGGCGCTTCTCAAGAGGAGTTCTACAAGTTCATCAAGCCGATCCTCGAGAAGATGGACGGCACCGAGGAGCTGCTCGTGAAGCTCAATAGAGAGGACCTCCTCCGCAAGCAGCGCACCTTCGATAATGGCTCCATCCCGCACCAGATCCACCTCGGCGAGCTTCATGCTATCCTCCGCAGGCAAGAGGACTTCTACCCGTTCCTCAAGGACAACCGCGAGAAGATTGAGAAGATCCTCACCTTCCGCATCCCGTACTACGTGGGCCCGCTCGCCAGGGGCAACTCCAGGTTCGCCTGGATGACCAGAAAGTCCGAGGAGACAATCACCCCCTGGAACTTCGAGGAGGTGGTGGATAAGGGCGCCTCTGCCCAGTCTTTCATCGAGCGCATGACCAACTTCGACAAGAACCTCCCGAACGAGAAGGTGCTCCCGAAGCACTCACTCCTCTACGAGTACTTCACCGTGTACAACGAGCTGACCAAGGTGAAGTACGTGACCGAGGGGATGAGGAAGCCAGCTTTCCTTAGCGGCGAGCAAAAGAAGGCCATCGTCGACCTGCTGTTCAAGACCAACCGCAAGGTGACCGTGAAGCAGCTCAAGGAGGACTACTTCAAGAAAATCGAGTGCTTCGACTCCGTCGAGATCTCCGGCGTCGAGGATAGGTTCAATGCCTCCCTCGGGACCTACCACGACCTCCTCAAGATTATCAAGGACAAGGACTTCCTCGACAACGAGGAGAACGAGGACATCCTCGAGGACATCGTGCTCACCCTCACCCTCTTCGAGGACCGCGAGATGATCGAGGAGCGCCTCAAGACATACGCCCACCTCTTCGACGACAAGGTGATGAAGCAGCTGAAGCGCAGGCGCTATACCGGCTGGGGCAGGCTCTCTAGGAAGCTCATCAACGGCATCCGCGACAAGCAGTCCGGCAAGACGATCCTCGACTTCCTCAAGTCCGACGGCTTCGCCAACCGCAACTTCATGCAGCTCATCCACGACGACTCCCTCACCTTCAAGGAGGACATCCAAAAGGCCCAGGTGTCCGGCCAAGGCGATTCCCTCCATGAGCATATCGCCAATCTCGCCGGCTCCCCGGCTATCAAGAAGGGCATTCTCCAGACCGTGAAGGTGGTGGACGAGCTGGTGAAGGTGATGGGCAGGCACAAGCCAGAGAACATCGTGATCGAGATGGCCCGCGAGAACCAGACCACACAGAAGGGCCAAAAGAACTCCCGCGAGCGCATGAAGAGGATCGAGGAGGGCATTAAGGAGCTGGGCTCCCAGATCCTCAAGGAGCACCCAGTCGAGAACACCCAGCTCCAGAACGAGAAGCTCTACCTCTACTACCTCCAGAACGGCCGCGACATGTACGTGGACCAAGAGCTGGACATCAACCGCCTCTCCGACTACGACGTGGACCATATTGTGCCGCAGTCCTTCCTGAAGGACGACTCCATCGACAACAAGGTGCTCACCCGCTCCGACAAGAACAGGGGCAAGTCCGATAACGTGCCGTCCGAAGAGGTCGTCAAGAAGATGAAGAACTACTGGCGCCAGCTCCTCAACGCCAAGCTCATCACCCAGAGGAAGTTCGACAACCTCACCAAGGCCGAGAGAGGCGGCCTTTCCGAGCTTGATAAGGCCGGCTTCATCAAGCGCCAGCTCGTCGAGACACGCCAGATCACAAAGCACGTGGCCCAGATCCTCGACTCCCGCATGAACACCAAGTACGACGAGAACGACAAGCTCATCCGCGAGGTGAAGGTCATCACCCTCAAGTCCAAGCTCGTGTCCGACTTCCGCAAGGACTTCCAGTTCTACAAGGTGCGCGAGATCAACAACTACCACCACGCCCACGACGCCTACCTCAATGCCGTGGTGGGCACAGCCCTCATCAAGAAGTACCCAAAGCTCGAGTCCGAGTTCGTGTACGGCGACTACAAGGTGTACGACGTGCGCAAGATGATCGCCAAGTCCGAGCAAGAGATCGGCAAGGCGACCGCCAAGTACTTCTTCTACTCCAACATCATGAATTTCTTCAAGACCGAGATCACGCTCGCCAACGGCGAGATTAGGAAGAGGCCGCTCATCGAGACAAACGGCGAGACAGGCGAGATCGTGTGGGACAAGGGCAGGGATTTCGCCACAGTGCGCAAGGTGCTCTCCATGCCGCAAGTGAACATCGTGAAGAAGACCGAGGTTCAGACCGGCGGCTTCTCCAAGGAGTCCATCCTCCCAAAGCGCAACTCCGACAAGCTGATCGCCCGCAAGAAGGACTGGGACCCGAAGAAGTATGGCGGCTTCCTCTGGCCGACCGTGGCCTACTCTGTGCTCGTGGTTGCCAAGGTCGAGAAGGGCAAGAGCAAGAAGCTCAAGTCCGTCAAGGAGCTGCTGGGCATCACGATCATGGAGCGCAGCAGCTTCGAGAAGAACCCAATCGACTTCCTCGAGGCCAAGGGCTACAAGGAGGTGAAGAAGGACCTCATCATCAAGCTCCCGAAGTACAGCCTCTTCGAGCTTGAGAACGGCCGCAAGAGAATGCTCGCCTCTGCTAAGCAGCTTCAGAAGGGCAACGAGCTTGCTCTCCCGTCCAAGTACGTGAACTTCCTCTACCTCGCCTCCCACTACGAGAAGCTCAAGGGCTCCCCAGAGGACAACGAGCAAAAGCAGCTGTTCGTCGAGCAGCACAAGCACTACCTCGACGAGATCATCGAGCAGATCTCCGAGTTCTCCAAGCGCGTGATCCTCGCCGATGCCAACCTCGATAAGGTGCTCAGCGCCTACAACAAGCACCGCGATAAGCCAATTCGCGAGCAGGCCGAGAACATCATCCACCTCTTCACCCTCACCAACCTCGGCGCTCCAGCCGCCTTCAAGTACTTCGACACCACCATCGACCGCAAGCAGTACCGCTCTACCAAGGAGGTTCTCGACGCCACCCTCATCCACCAGTCTATCACAGGCCTCTACGAGACACGCATCGACCTCTCACAACTCGGCGGCGATTCCGGCGGCAGCCCAAAGAAGAAGCGGAAGGTGTCTGGAGGTTCTCCTAAGAAAAAGAGAAAAGTGTCCGGCGGCTCCCCGAAGAAGAAGCGCAAGGTGTGA |
| SpRY | ATGGCTCCGAAGAAGAAGAGGAAGGTTGGCATCCACGGGGTGCCAGCTGCTGACAAGAAGTACTCCATCGGCCTCGACATCGGCACCAATTCTGTTGGCTGGGCCGTGATCACCGACGAGTACAAGGTGCCGTCCAAGAAGTTCAAGGTCCTCGGCAACACCGACCGCCACTCCATCAAGAAGAATCTCATCGGCGCCCTGCTGTTCGACTCTGGCGAGACAGCCGAGCGTACAAGGCTCAAGAGGACCGCTAGACGCAGGTACACCAGGCGCAAGAACCGCATCTGCTACCTCCAAGAGATCTTCTCCAACGAGATGGCCAAGGTGGACGACAGCTTCTTCCACAGGCTCGAGGAGAGCTTCCTCGTCGAGGAGGACAAGAAGCACGAGCGCCATCCGATCTTCGGCAACATCGTGGATGAGGTGGCCTACCACGAGAAGTACCCGACCATCTACCACCTCCGCAAGAAGCTCGTCGACTCCACCGATAAGGCCGACCTCAGGCTCATCTACCTCGCCCTCGCCCACATGATCAAGTTCAGGGGCCACTTCCTCATCGAGGGCGACCTCAACCCGGACAACTCCGATGTGGACAAGCTGTTCATCCAGCTCGTGCAGACCTACAACCAGCTGTTCGAGGAGAACCCGATCAACGCCTCTGGCGTTGACGCCAAGGCTATTCTCTCTGCCAGGCTCTCTAAGTCCCGCAGGCTCGAGAATCTGATCGCCCAACTTCCGGGCGAGAAGAAGAATGGCCTCTTCGGCAACCTGATCGCCCTCTCTCTTGGCCTCACCCCGAACTTCAAGTCCAACTTCGACCTCGCCGAGGACGCCAAGCTCCAGCTTTCCAAGGACACCTACGACGACGACCTCGACAATCTCCTCGCCCAGATTGGCGATCAGTACGCCGATCTGTTCCTCGCCGCCAAGAATCTCTCCGACGCCATCCTCCTCAGCGACATCCTCAGGGTGAACACCGAGATCACCAAGGCCCCACTCTCCGCCTCCATGATCAAGAGGTACGACGAGCACCACCAGGACCTCACACTCCTCAAGGCCCTCGTGAGACAGCAGCTCCCAGAGAAGTACAAGGAGATCTTCTTCGACCAGTCCAAGAACGGCTACGCCGGCTACATCGATGGCGGCGCTTCTCAAGAGGAGTTCTACAAGTTCATCAAGCCGATCCTCGAGAAGATGGACGGCACCGAGGAGCTGCTCGTGAAGCTCAATAGAGAGGACCTCCTCCGCAAGCAGCGCACCTTCGATAATGGCTCCATCCCGCACCAGATCCACCTCGGCGAGCTTCATGCTATCCTCCGCAGGCAAGAGGACTTCTACCCGTTCCTCAAGGACAACCGCGAGAAGATTGAGAAGATCCTCACCTTCCGCATCCCGTACTACGTGGGCCCGCTCGCCAGGGGCAACTCCAGGTTCGCCTGGATGACCAGAAAGTCCGAGGAGACAATCACCCCCTGGAACTTCGAGGAGGTGGTGGATAAGGGCGCCTCTGCCCAGTCTTTCATCGAGCGCATGACCAACTTCGACAAGAACCTCCCGAACGAGAAGGTGCTCCCGAAGCACTCACTCCTCTACGAGTACTTCACCGTGTACAACGAGCTGACCAAGGTGAAGTACGTGACCGAGGGGATGAGGAAGCCAGCTTTCCTTAGCGGCGAGCAAAAGAAGGCCATCGTCGACCTGCTGTTCAAGACCAACCGCAAGGTGACCGTGAAGCAGCTCAAGGAGGACTACTTCAAGAAAATCGAGTGCTTCGACTCCGTCGAGATCTCCGGCGTCGAGGATAGGTTCAATGCCTCCCTCGGGACCTACCACGACCTCCTCAAGATTATCAAGGACAAGGACTTCCTCGACAACGAGGAGAACGAGGACATCCTCGAGGACATCGTGCTCACCCTCACCCTCTTCGAGGACCGCGAGATGATCGAGGAGCGCCTCAAGACATACGCCCACCTCTTCGACGACAAGGTGATGAAGCAGCTGAAGCGCAGGCGCTATACCGGCTGGGGCAGGCTCTCTAGGAAGCTCATCAACGGCATCCGCGACAAGCAGTCCGGCAAGACGATCCTCGACTTCCTCAAGTCCGACGGCTTCGCCAACCGCAACTTCATGCAGCTCATCCACGACGACTCCCTCACCTTCAAGGAGGACATCCAAAAGGCCCAGGTGTCCGGCCAAGGCGATTCCCTCCATGAGCATATCGCCAATCTCGCCGGCTCCCCGGCTATCAAGAAGGGCATTCTCCAGACCGTGAAGGTGGTGGACGAGCTGGTGAAGGTGATGGGCAGGCACAAGCCAGAGAACATCGTGATCGAGATGGCCCGCGAGAACCAGACCACACAGAAGGGCCAAAAGAACTCCCGCGAGCGCATGAAGAGGATCGAGGAGGGCATTAAGGAGCTGGGCTCCCAGATCCTCAAGGAGCACCCAGTCGAGAACACCCAGCTCCAGAACGAGAAGCTCTACCTCTACTACCTCCAGAACGGCCGCGACATGTACGTGGACCAAGAGCTGGACATCAACCGCCTCTCCGACTACGACGTGGACCATATTGTGCCGCAGTCCTTCCTGAAGGACGACTCCATCGACAACAAGGTGCTCACCCGCTCCGACAAGAACAGGGGCAAGTCCGATAACGTGCCGTCCGAAGAGGTCGTCAAGAAGATGAAGAACTACTGGCGCCAGCTCCTCAACGCCAAGCTCATCACCCAGAGGAAGTTCGACAACCTCACCAAGGCCGAGAGAGGCGGCCTTTCCGAGCTTGATAAGGCCGGCTTCATCAAGCGCCAGCTCGTCGAGACACGCCAGATCACAAAGCACGTGGCCCAGATCCTCGACTCCCGCATGAACACCAAGTACGACGAGAACGACAAGCTCATCCGCGAGGTGAAGGTCATCACCCTCAAGTCCAAGCTCGTGTCCGACTTCCGCAAGGACTTCCAGTTCTACAAGGTGCGCGAGATCAACAACTACCACCACGCCCACGACGCCTACCTCAATGCCGTGGTGGGCACAGCCCTCATCAAGAAGTACCCAAAGCTCGAGTCCGAGTTCGTGTACGGCGACTACAAGGTGTACGACGTGCGCAAGATGATCGCCAAGTCCGAGCAAGAGATCGGCAAGGCGACCGCCAAGTACTTCTTCTACTCCAACATCATGAATTTCTTCAAGACCGAGATCACGCTCGCCAACGGCGAGATTAGGAAGAGGCCGCTCATCGAGACAAACGGCGAGACAGGCGAGATCGTGTGGGACAAGGGCAGGGATTTCGCCACAGTGCGCAAGGTGCTCTCCATGCCGCAAGTGAACATCGTGAAGAAGACCGAGGTTCAGACCGGCGGCTTCTCCAAGGAGTCCATCCGCCCAAAGCGCAACTCCGACAAGCTGATCGCCCGCAAGAAGGACTGGGACCCGAAGAAGTATGGCGGCTTCCTCTGGCCGACCGTGGCCTACTCTGTGCTCGTGGTTGCCAAGGTCGAGAAGGGCAAGAGCAAGAAGCTCAAGTCCGTCAAGGAGCTGCTGGGCATCACGATCATGGAGCGCAGCAGCTTCGAGAAGAACCCAATCGACTTCCTCGAGGCCAAGGGCTACAAGGAGGTGAAGAAGGACCTCATCATCAAGCTCCCGAAGTACAGCCTCTTCGAGCTTGAGAACGGCCGCAAGAGAATGCTCGCCTCTGCTAAGCAGCTTCAGAAGGGCAACGAGCTTGCTCTCCCGTCCAAGTACGTGAACTTCCTCTACCTCGCCTCCCACTACGAGAAGCTCAAGGGCTCCCCAGAGGACAACGAGCAAAAGCAGCTGTTCGTCGAGCAGCACAAGCACTACCTCGACGAGATCATCGAGCAGATCTCCGAGTTCTCCAAGCGCGTGATCCTCGCCGATGCCAACCTCGATAAGGTGCTCAGCGCCTACAACAAGCACCGCGATAAGCCAATTCGCGAGCAGGCCGAGAACATCATCCACCTCTTCACCCTCACCCGCCTCGGCGCTCCACGCGCCTTCAAGTACTTCGACACCACCATCGACCCCAAGCAGTACCGCTCTACCAAGGAGGTTCTCGACGCCACCCTCATCCACCAGTCTATCACAGGCCTCTACGAGACACGCATCGACCTCTCACAACTCGGCGGCGATAAGCGGCCAGCGGCGACGAAGAAGGCGGGGCAGGCGAAGAAGAAGAAGTGA |
| SpRY-ABE8e | ATGGCTCCGAAGAAGAAGAGGAAGGTTGGCATCCACGGGGTGCCAGCTGCTAGCGAGGTGGAGTTCAGCCACGAGTACTGGATGAGGCATGCCCTCACACTCGCAAAGAGGGCGCGCGATGAGAGGGAGGTGCCTGTCGGCGCGGTGCTCGTCCTGAACAATCGCGTGATCGGAGAGGGATGGAACCGGGCAATTGGCCTCCATGACCCAACAGCACATGCCGAGATCATGGCCCTCAGGCAGGGCGGCCTGGTCATGCAGAATTACCGGCTCATTGATGCCACCCTCTACGTGACATTCGAGCCATGCGTCATGTGCGCGGGAGCCATGATCCACTCACGGATTGGCAGGGTGGTCTTCGGAGTGAGGAACTCAAAGCGCGGCGCCGCGGGCTCTCTCATGAACGTGCTGAATTACCCAGGCATGAATCATCGGGTCGAGATCACAGAGGGCATTCTGGCGGATGAGTGCGCAGCCCTCCTGTGCGATTTCTACCGCATGCCTCGGCAGGTCTTCAACGCCCAGAAGAAGGCGCAGTCCAGCATCAATTCCGGCGGCTCATCTGGCGGCTCCAGCGGCAGCGAGACACCAGGCACATCAGAGTCTGCGACACCGGAGTCATCTGGCGGGTCCTCCGGTGGTTCCGACAAGAAGTACTCCATCGGCCTCGCTATCGGCACCAATTCTGTTGGCTGGGCCGTGATCACCGACGAGTACAAGGTGCCGTCCAAGAAGTTCAAGGTCCTCGGCAACACCGACCGCCACTCCATCAAGAAGAATCTCATCGGCGCCCTGCTGTTCGACTCTGGCGAGACAGCCGAGCGTACAAGGCTCAAGAGGACCGCTAGACGCAGGTACACCAGGCGCAAGAACCGCATCTGCTACCTCCAAGAGATCTTCTCCAACGAGATGGCCAAGGTGGACGACAGCTTCTTCCACAGGCTCGAGGAGAGCTTCCTCGTCGAGGAGGACAAGAAGCACGAGCGCCATCCGATCTTCGGCAACATCGTGGATGAGGTGGCCTACCACGAGAAGTACCCGACCATCTACCACCTCCGCAAGAAGCTCGTCGACTCCACCGATAAGGCCGACCTCAGGCTCATCTACCTCGCCCTCGCCCACATGATCAAGTTCAGGGGCCACTTCCTCATCGAGGGCGACCTCAACCCGGACAACTCCGATGTGGACAAGCTGTTCATCCAGCTCGTGCAGACCTACAACCAGCTGTTCGAGGAGAACCCGATCAACGCCTCTGGCGTTGACGCCAAGGCTATTCTCTCTGCCAGGCTCTCTAAGTCCCGCAGGCTCGAGAATCTGATCGCCCAACTTCCGGGCGAGAAGAAGAATGGCCTCTTCGGCAACCTGATCGCCCTCTCTCTTGGCCTCACCCCGAACTTCAAGTCCAACTTCGACCTCGCCGAGGACGCCAAGCTCCAGCTTTCCAAGGACACCTACGACGACGACCTCGACAATCTCCTCGCCCAGATTGGCGATCAGTACGCCGATCTGTTCCTCGCCGCCAAGAATCTCTCCGACGCCATCCTCCTCAGCGACATCCTCAGGGTGAACACCGAGATCACCAAGGCCCCACTCTCCGCCTCCATGATCAAGAGGTACGACGAGCACCACCAGGACCTCACACTCCTCAAGGCCCTCGTGAGACAGCAGCTCCCAGAGAAGTACAAGGAGATCTTCTTCGACCAGTCCAAGAACGGCTACGCCGGCTACATCGATGGCGGCGCTTCTCAAGAGGAGTTCTACAAGTTCATCAAGCCGATCCTCGAGAAGATGGACGGCACCGAGGAGCTGCTCGTGAAGCTCAATAGAGAGGACCTCCTCCGCAAGCAGCGCACCTTCGATAATGGCTCCATCCCGCACCAGATCCACCTCGGCGAGCTTCATGCTATCCTCCGCAGGCAAGAGGACTTCTACCCGTTCCTCAAGGACAACCGCGAGAAGATTGAGAAGATCCTCACCTTCCGCATCCCGTACTACGTGGGCCCGCTCGCCAGGGGCAACTCCAGGTTCGCCTGGATGACCAGAAAGTCCGAGGAGACAATCACCCCCTGGAACTTCGAGGAGGTGGTGGATAAGGGCGCCTCTGCCCAGTCTTTCATCGAGCGCATGACCAACTTCGACAAGAACCTCCCGAACGAGAAGGTGCTCCCGAAGCACTCACTCCTCTACGAGTACTTCACCGTGTACAACGAGCTGACCAAGGTGAAGTACGTGACCGAGGGGATGAGGAAGCCAGCTTTCCTTAGCGGCGAGCAAAAGAAGGCCATCGTCGACCTGCTGTTCAAGACCAACCGCAAGGTGACCGTGAAGCAGCTCAAGGAGGACTACTTCAAGAAAATCGAGTGCTTCGACTCCGTCGAGATCTCCGGCGTCGAGGATAGGTTCAATGCCTCCCTCGGGACCTACCACGACCTCCTCAAGATTATCAAGGACAAGGACTTCCTCGACAACGAGGAGAACGAGGACATCCTCGAGGACATCGTGCTCACCCTCACCCTCTTCGAGGACCGCGAGATGATCGAGGAGCGCCTCAAGACATACGCCCACCTCTTCGACGACAAGGTGATGAAGCAGCTGAAGCGCAGGCGCTATACCGGCTGGGGCAGGCTCTCTAGGAAGCTCATCAACGGCATCCGCGACAAGCAGTCCGGCAAGACGATCCTCGACTTCCTCAAGTCCGACGGCTTCGCCAACCGCAACTTCATGCAGCTCATCCACGACGACTCCCTCACCTTCAAGGAGGACATCCAAAAGGCCCAGGTGTCCGGCCAAGGCGATTCCCTCCATGAGCATATCGCCAATCTCGCCGGCTCCCCGGCTATCAAGAAGGGCATTCTCCAGACCGTGAAGGTGGTGGACGAGCTGGTGAAGGTGATGGGCAGGCACAAGCCAGAGAACATCGTGATCGAGATGGCCCGCGAGAACCAGACCACACAGAAGGGCCAAAAGAACTCCCGCGAGCGCATGAAGAGGATCGAGGAGGGCATTAAGGAGCTGGGCTCCCAGATCCTCAAGGAGCACCCAGTCGAGAACACCCAGCTCCAGAACGAGAAGCTCTACCTCTACTACCTCCAGAACGGCCGCGACATGTACGTGGACCAAGAGCTGGACATCAACCGCCTCTCCGACTACGACGTGGACCATATTGTGCCGCAGTCCTTCCTGAAGGACGACTCCATCGACAACAAGGTGCTCACCCGCTCCGACAAGAACAGGGGCAAGTCCGATAACGTGCCGTCCGAAGAGGTCGTCAAGAAGATGAAGAACTACTGGCGCCAGCTCCTCAACGCCAAGCTCATCACCCAGAGGAAGTTCGACAACCTCACCAAGGCCGAGAGAGGCGGCCTTTCCGAGCTTGATAAGGCCGGCTTCATCAAGCGCCAGCTCGTCGAGACACGCCAGATCACAAAGCACGTGGCCCAGATCCTCGACTCCCGCATGAACACCAAGTACGACGAGAACGACAAGCTCATCCGCGAGGTGAAGGTCATCACCCTCAAGTCCAAGCTCGTGTCCGACTTCCGCAAGGACTTCCAGTTCTACAAGGTGCGCGAGATCAACAACTACCACCACGCCCACGACGCCTACCTCAATGCCGTGGTGGGCACAGCCCTCATCAAGAAGTACCCAAAGCTCGAGTCCGAGTTCGTGTACGGCGACTACAAGGTGTACGACGTGCGCAAGATGATCGCCAAGTCCGAGCAAGAGATCGGCAAGGCGACCGCCAAGTACTTCTTCTACTCCAACATCATGAATTTCTTCAAGACCGAGATCACGCTCGCCAACGGCGAGATTAGGAAGAGGCCGCTCATCGAGACAAACGGCGAGACAGGCGAGATCGTGTGGGACAAGGGCAGGGATTTCGCCACAGTGCGCAAGGTGCTCTCCATGCCGCAAGTGAACATCGTGAAGAAGACCGAGGTTCAGACCGGCGGCTTCTCCAAGGAGTCCATCCGCCCAAAGCGCAACTCCGACAAGCTGATCGCCCGCAAGAAGGACTGGGACCCGAAGAAGTATGGCGGCTTCCTCTGGCCGACCGTGGCCTACTCTGTGCTCGTGGTTGCCAAGGTCGAGAAGGGCAAGAGCAAGAAGCTCAAGTCCGTCAAGGAGCTGCTGGGCATCACGATCATGGAGCGCAGCAGCTTCGAGAAGAACCCAATCGACTTCCTCGAGGCCAAGGGCTACAAGGAGGTGAAGAAGGACCTCATCATCAAGCTCCCGAAGTACAGCCTCTTCGAGCTTGAGAACGGCCGCAAGAGAATGCTCGCCTCTGCTAAGCAGCTTCAGAAGGGCAACGAGCTTGCTCTCCCGTCCAAGTACGTGAACTTCCTCTACCTCGCCTCCCACTACGAGAAGCTCAAGGGCTCCCCAGAGGACAACGAGCAAAAGCAGCTGTTCGTCGAGCAGCACAAGCACTACCTCGACGAGATCATCGAGCAGATCTCCGAGTTCTCCAAGCGCGTGATCCTCGCCGATGCCAACCTCGATAAGGTGCTCAGCGCCTACAACAAGCACCGCGATAAGCCAATTCGCGAGCAGGCCGAGAACATCATCCACCTCTTCACCCTCACCCGCCTCGGCGCTCCACGCGCCTTCAAGTACTTCGACACCACCATCGACCCCAAGCAGTACCGCTCTACCAAGGAGGTTCTCGACGCCACCCTCATCCACCAGTCTATCACAGGCCTCTACGAGACACGCATCGACCTCTCACAACTCGGCGGCGATAAGCGGCCAGCGGCGACGAAGAAGGCGGGGCAGGCGAAGAAGAAGAAGTGA |
